# Coexistence of genetically different *Rhizophagus irregularis* isolates induces genes involved in a putative fungal mating response

**DOI:** 10.1038/s41396-020-0694-3

**Published:** 2020-06-08

**Authors:** Ivan D. Mateus, Edward C. Rojas, Romain Savary, Cindy Dupuis, Frédéric G. Masclaux, Consolée Aletti, Ian R. Sanders

**Affiliations:** Department of Ecology and Evolution, University of Lausanne, Biophore building, 1015 Lausanne, Switzerland

**Keywords:** Ecology, Fungi

## Abstract

Arbuscular mycorrhizal fungi (AMF) are of great ecological importance because of their effects on plant growth. Closely related genotypes of the same AMF species coexist in plant roots. However, almost nothing is known about the molecular interactions occurring during such coexistence. We compared *in planta* AMF gene transcription in single and coinoculation treatments with two genetically different isolates of *Rhizophagus irregularis* in symbiosis independently on three genetically different cassava genotypes. Remarkably few genes were specifically upregulated when the two fungi coexisted. Strikingly, almost all of the genes with an identifiable putative function were known to be involved in mating in other fungal species. Several genes were consistent across host plant genotypes but more upregulated genes involved in putative mating were observed in host genotype (COL2215) compared with the two other host genotypes. The AMF genes that we observed to be specifically upregulated during coexistence were either involved in the mating pheromone response, in meiosis, sexual sporulation or were homologs of MAT-locus genes known in other fungal species. We did not observe the upregulation of the expected homeodomain genes contained in a putative AMF MAT-locus, but observed upregulation of HMG-box genes similar to those known to be involved in mating in Mucoromycotina species. Finally, we demonstrated that coexistence between the two fungal genotypes in the coinoculation treatments explained the number of putative mating response genes activated in the different plant host genotypes. This study demonstrates experimentally the activation of genes involved in a putative mating response and represents an important step towards the understanding of coexistence and sexual reproduction in these important plant symbionts.

## Introduction

Most terrestrial plants form symbioses with arbuscular mycorrhizal fungi (AMF), and this greatly impacts plant growth and diversity of plant communities [1, 2]. These fungi, representing the subphylum Glomeromycotina [3], have formed symbioses with plants for more than 450 million years [4]. In nature, different genotypes of the same AMF species co-occur in plants and are, thus, faced with competing for the same root-space. Different genotypes of *R. irregularis* are known to individually differentially affect plant growth [5]. However, the coexistence between the different genotypes within a plant is strongly influenced by their genetic distance and impacts plant growth [6]. It is, therefore, highly relevant to understand the molecular interactions that occur between closely related but genetically different coexisting *R. irregularis* genotypes within plant roots. Such interactions could potentially range from the transcription of genes that allow recognition between pairs of fungal genotypes to more direct interactions leading to sexual reproduction; a process that probably remains highly elusive to researchers because it may be very rare [7]. To date, we are not aware of any studies that have looked at such molecular interactions between AMF genotypes *in planta*.

Despite their enormous biological importance, the complete life cycle and mode of reproduction in AMF is still not well understood [8]. No evidence of sexual structures has ever been recorded in AMF, and they have long been considered as ancient asexuals [9]. Anastomosis, cytoplasmic exchange, and streaming have been shown between hyphae of the same fungal isolate [10] and between hyphae of genetically different isolates of the same fungal species [11–13]. It has been suggested that the multinucleated nature [14] and anastomosis [15] represent mechanisms of maintenance of genetic diversity in the absence of sexual recombination. AMF clearly reproduce clonally much of the time as identical genotypes occur over very large geographical distances indicating sexual reproduction may be very rare [16]. However, the notion of complete asexuality in these taxa is unlikely because: (1) a full repertoire of genes required for meiosis is present in the genome of the model AM fungus *R. irregularis* [17, 18]; (2) a putative MAT-locus has been proposed and identified in different Glomerales and Diversiporales species [19]; (3) population genetic data suggest the existence of recombination in AM fungus populations [20]; (4) some internucleus recombination has been reported [21], although the existence of adequate evidence to support this has recently been questioned [22]. Sexual reproduction in AMF may, indeed, occur frequently enough to purge deleterious mutations, but may not be frequent enough to be readily observable in experiments, making its experimental observation highly elusive [7].

The genomic region that defines mating compatibility and sexual reproduction in fungi (MAT-locus) is not fully conserved among phyla. At least three different transcription factors (homeodomain (HD), alpha-box, and HMG-box) are responsible for sex-determination in different fungal phyla [23–29]. It has been suggested that the fungal ancestral MAT-locus contained an HMG-box transcription factor. This is supported by its presence in Zygomycetes, Microsporidia, Hemiascomycetes, and Euascomycetes. However, in Ascomycetes and Basidiomycetes, HMG-box transcription factors are replaced by alpha-box and HD transcription factors [29]. In Mucoromycotina, a closely related subphylum to AMF [30], the MAT-locus comprises an HMG-box transcription factor containing an HMG-box domain located between two flanking genes that code for a triose-phosphate transporter (TPT) homolog and a helicase. However, the presence of additional genes is possible as in *P. blakesleeanus* and in *R. oryzae* [26]. Two different forms of the HMG-box transcription factor (SexM and SexP) exist, but each individual only carries one of them. Exceptions to the rule also occur as in the homothallic fungus *Syzygites megalocarpus* [31] where an individual contains two MAT-loci each containing a SexP and SexM copy.

When two individuals of the same species interact, nonself-recognition is initiated by the perception of mating pheromones. The presence of mating pheromones activates at least four different mitogen-activated protein kinase (MAPK) cascades during the mating process [32]. Activation of MAT-locus sex-determination transcription factors is also expected [33, 34]. This process leads to the formation of the mating tube and the induction of meiosis and sexual sporulation.

Despite the likely existence of sexual processes in AMF, the molecular mechanisms of mating in these fungi are poorly understood. Homologs of genes present in the MAT-locus of closely related Mucoromycotina species were found in *R. irregularis*. However, they are not contiguous, and are located in different regions of the genome [35]. No activation of HMG-box genes was detected when two genetically different *R. irregularis* isolates were co-cultured in vitro [36]. A study of *R. irregularis* isolates reported the identification of a putative MAT-locus, based on homology, that contained an HD1-like and HD2 HD gene in opposite transcriptional directions [8]. The presence of this locus was confirmed in the Glomerales and Diversiporales, with the exception of Gigasporaceae, where the locus presented no structural conservation [19]. However, no study has provided any experimental evidence of the involvement of this putative MAT-locus, or any other genes, in the interaction between genetically different AMF isolates of the same species. Curiously, no studies have ever tried to look for evidence of transcription when isolates of the same AMF species coexist *in planta*, even though it is known that genetically different isolates of AMF co-colonize roots.

In this study, we investigated the molecular mechanisms of nonself-interactions between two genetically different, but closely related, *R. irregularis* isolates coexisting *in planta*. We compared gene transcription of two genetically different *R. irregularis* isolates, either colonizing roots alone or when the two fungi coexisted in cassava (*Manihot esculenta* Cranz) roots (Supplementary Fig. 1 and Supplementary Note 1). First, we tested if fungal genes were differentially transcribed in the coinoculation treatment compared with both single-inoculation treatments. Second, we evaluated if there were different fungal transcriptional response in the three host genotypes. Third, we evaluated if the proportion of colonization by each isolate was different among the three host plant genotypes in the coinoculation treatments. Understanding the molecular mechanisms of nonself-interactions is highly relevant in the understanding how AMF interact with each other in plant roots. Identifying which genes are activated when two AMF meet, and if this occurs in symbiosis with different host plant genetic backgrounds, will help identify the molecular mechanisms of AM fungal coexistence, give clues about AMF nonself-recognition and how it is affected by the genetic background of host plants.

## Methods

### Biological material and growth conditions

We conducted a greenhouse experiment where cassava (*M. esculenta*) was inoculated singly with two different *R. irregularis* isolates independently, coinoculated with the two isolates together or mock-inoculated. We performed this experiment independently on three different cassava (*M. esculenta*) genotypes (cultivars COL2215, BRA337, and CM4574). The cassava cultivars were obtained from the cassava genebank resource at the International Center for Tropical Agriculture and two *R. irregularis* isolates (B1 and DAOM197198), originally collected from Tänikon, Switzerland and Pont Rouge, Canada, respectively. This experiment was run in parallel with another experiment designed to answer a separate question regarding how cassava genotypes affected cassava and *R. irregularis* gene transcription in symbiosis [37]. The phenotypic data on single-inoculations and the mock-inoculated samples was already published in the previous publication. The RNA-seq data on the single-inoculation samples and the mock-inoculated treatments were already made publicly available under the accession number PRJNA400637. The fungal colonization, plant response, and transcript data of the coinoculation treatments have not previously been published. Sequence reads were deposited in the NCBI SRA database (BioProject: PRJNA494798). The source code describing the steps of the bioinformatic analysis and the differential gene transcription analysis can be found in Supplementary File 1.

We performed a greenhouse experiment by inoculating micropropagated cassava plants with spores produced in in vitro cultures (Supplementary Note 2). The methods concerning the fungal colonization and plant growth responses can be found in Supplementary Note 3.

### RNA extraction, library preparation, sequencing, and bioinformatic analysis

The RNA extraction, library preparation, sequencing, and the bioinformatic analysis were performed exactly according to a previously published dual RNA-seq analysis experiment [37] (Supplementary Note 4).

### Sequence containment estimation on raw data

In order to test for contamination by other fungi in the RNA sequencing data set, we estimated the presence of different fungal taxa in the raw sequencing reads with the Mash screen algorithm [38]. We first downloaded a single genome assembly for every fungal species present in the RefSeq NCBI database, resulting in 1721 different genome assemblies. We used the Mash sketch algorithm to create a combined reference file containing the 1721 different genomes. We screened each sample raw sequencing reads against the reference file by using the Mash screen algorithm with the winner-take-all strategy. To illustrate these results, we produced a heatmap in R.

### Differential transcription analysis

We used the DEseq2 pipeline for detecting differential gene transcription among treatments [39]. We used the raw read count data tables from feature Counts as input. We constructed the DEseq data set by including the read count tables per sample and the different conditions to be evaluated. We then performed the default DESeq differential transcription analysis based on a negative-binomial distribution and producing Wald statistics. The DEseq pipeline performed an internal normalization method that corrects for library size and RNA composition bias. In addition, it uses a false discovery rate method “BH” to adjust the probability (*P*) for multiple comparisons [39].

Previously, genetically different plant genotypes have shown to strongly affect fungal gene transcription [37]. Therefore, we independently analyzed the data for each plant host genotype, to avoid the effect of plant genotype variability on fungal gene transcription. We only report genes that displayed significantly different transcript levels (*adjusted p* < 0.1) for both comparisons: (1) between the single-inoculation treatment with isolate DAOM197198 and the coinoculation treatment and (2) between the single-inoculation treatment with isolate B1 and the coinoculation treatment. For illustration purposes, we showed the normalized counts of all treatments together and not the pairwise comparisons.

### SNP analysis of RNA-seq data

We called SNPs from the BAM files with the software freebayes 1.2.0 [40], using the following parameters: ploidy equal to 1, minimum mapping quality of 30 and minimum coverage of 10 reads. We produced a single variant calling VCF file for each sample and we analyzed the inoculated samples (single-inoculations and coinoculations) for each cultivar independently. We merged the different VCF files with the BCFtools merge option included in the samtools suite [41]. We then filtered and kept: (1) the positions where SNPs were detected in at least one sample. (2) The positions where all the samples displayed a call (no missing data). (3) The positions where the B1 samples displayed a SNP (GT:1). We then measured the identity and frequency of the reference and alternative allele of each position by using the fields genotype (GT) and depth (DP) of the VCF file. The allele frequency estimation and displays were performed in R and with the ggplot2 package.

### Conserved domain extraction and phylogenetic analyses

We extracted the conserved domains of genes from the NCBI conserved domain database (CDD) by using the “align detail” output of the Batch CD-search tool [42]. The alignment of the sequences was made using the MAFFT v7 online version with the “auto” option [43]. The alignment figures were generated by the Expasy Boxshade v3.21 online tool. We reconstructed the phylogenetic relationship among sequences by first identifying the most fitted substitution model of the sequences with MEGA X software [44]. We then performed maximum likelihood phylogenies on gap-free sites, and we used bootstraps of 100 resamplings. The phylogenies were constructed with MEGA X and the figures were generated using R packages “ape” [45] and “ggtree” [46].

### Functional annotation and comparative genomics analyses

We blasted the upregulated genes against the NCBI nonredundant protein sequence database to find gene identity [47]. In addition, we identified homologs on fungal model species by doing a reciprocal blasting to genes observed in model fungal organisms as *Saccharomyces cerevisiae*, *Schizosaccharomyces pombe*, *Aspergillus nidulans*, *Ustilago maydis*, and *Mucor circinelloides*. We used CD-Search of the CDD to identify the conserved domains in the gene sequences.

We used published available *R. irregularis* genome assemblies of isolates DAOM197198 (GCA_002897155.1), A1 (GCA_001593125.1), A5 (GCA_001593205.1), A4 (GCA_001593145.1), and C2 (GCA_001593155.1) to identify the locus of the HMG-box MAT-encodes homolog genes. We used the console NCBI app blast functions to identify homologous genes between the different genomes assemblies and used Easyfig [48] for the synteny visualization.

## Results

### RNA-seq data quality

We obtained an average of 42 million reads per sample, from which 33.8 million reads uniquely mapped to the cassava genome. Six million reads did not map to the cassava genome and 3.45 million reads mapped to the *R. irregularis* genome (Supplementary Fig. 2 and Supplementary File 2). We did not observe any statistically significant difference in the number of total reads, reads uniquely mapped to cassava, reads uniquely mapped to *R. irregularis*, and the number of genes detected between the coinoculation treatment compared with each single-inoculation treatment in plant genotype COL2215. However, we found that the total number of reads differed between the coinoculation treatment and both single-inoculation treatments in plant genotype CM4574. We also observed that the number of genes that were sequenced differed between the coinoculation and DAOM197198 isolate on plant genotype BRA337. (Supplementary File 2). In addition, a detailed analysis of data quality and its robustness were previously shown for the mock and single-inoculation treatments in Mateus et al. [37].

We observed a negligible number of fungal transcripts in the mock-inoculated plants and a large number of fungal transcripts in the fungal inoculated plants in all three cultivars and in all fungal inoculated treatments. This showed that *R. irregularis* was present in the single-inoculation and coinoculation treatments and that the method was robust for distinguishing fungal transcripts from cassava transcripts (Supplementary Fig. 2 and Supplementary File 2) [37]. We also observed that the coinoculation treatment resulted in distinct patterns of fungal gene transcription from either of the single inoculations (Supplementary Fig. 3).

We analyzed the raw reads for the presence of fungal sequences that could have contaminated the plants in the greenhouse. We identified only *R. irregularis* sequences in the inoculation samples. Furthermore, we did not find any fungal sequences in the mock-inoculated samples. Taken together, these results mean that the data from inoculated plants only contained transcripts of *R. irregularis* and not any contaminating fungi (Supplementary Fig. 4 and Supplementary File 3).

### Fungal colonization and plant growth responses

We observed that the mock-inoculated samples did not display any fungal colonization. We also observed no statistically significant differences in the colonization rates between the coinoculation and the single-inoculation treatments on any plant genotype (Supplementary Fig. 5 and Supplementary File 4). We did not observe any significant differences in plant growth in any host plant genotype between the two single-inoculation and the coinoculation treatments (Supplementary File 5). We observed significant differences in aboveground dry weight between the coinoculation and the DAOM197198 isolate in plant genotype COL2215 (Supplementary Fig. 6a). We did not observe any significant differences in growth between the coinoculation and the single-inoculation treatments in plant genotype CM4574 (Supplementary Fig. 6b). There were significant differences in aboveground and total dry weight between the coinoculation and the DAOM197198 isolate in plant genotype BRA337 (Supplementary Fig. 6c).

### HMG-box homologs of Mucoromycotina SexM MAT-locus are upregulated in the coinoculation treatment

**Fig. 1:**
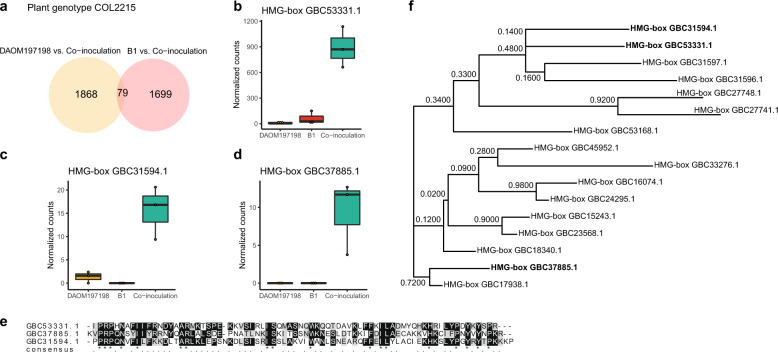
Differential gene transcription in host COL2215 reveals upregulation of HMG-box genes in the co-inoculation treatment. **a** Results of the analysis of *R. irregularis* differential gene transcription in host genotype COL2215 and comparing single-inoculation treatments to the coinoculation treatment. **b** Transcription of the *R. irregularis* HMG-box gene GBC53331.1. **c** Transcription of the *R. irregularis* HMG-box gene GBC31594.1. **d** Transcription of the *R. irregularis* HMG-box gene GBC37885.1 in cassava genotype COL2215. Normalized counts of transcripts that displayed significantly higher gene transcription in the coinoculation treatment (with isolates B1 and DAOM197198) compared with the two single-inoculation treatments (B1 or DAOM197198). **e** Sequence alignment of HMG-box conserved domain of the three upregulated *R. irregularis* HMG-box genes. **f** Phylogenetic reconstruction using LG-G as substitution model and 100 bootstraps of the HMG-box conserved domain of a subset of HMG-box genes found in *R. irregularis* DAOM197198 genome assembly. The three upregulated HMG-box genes are shown in bold type.

We observed 79 genes that displayed a significantly different level of transcription in the coinoculation treatment compared with the two single-inoculation treatments when associated with the host plant genotype COL2215 (Fig. 1a and Supplementary File 6). Most of the upregulated genes represented proteins of unknown function. However, of the genes with a known function, we identified three different HMG-box genes, containing an HMG-box domain: GBC53331.1, GBC31594.1, and GBC37885.1 (Fig. 1b–d), that were specifically upregulated in the coinoculation treatment in host genotype COL2215. We compared the amino acid sequence of the HMG-box conserved domain in a subset of HMG-box genes containing the three differentially transcribed HMG-box genes and observed that GBC53331.1 and GBC31594.1 shared a more similar amino acid sequence compared with GBC37885.1 (Fig. 1e, f).

Previous studies in several fungi have shown that genes containing an HMG-box domain are involved in fungal mating in two different ways: (1) they represent part of the MAT-locus [49] or (2) they are involved in the pheromone response during the recognition and mating process (i.e. Prf1 in *U. maydis*, Mat2 in *Cryptococcus neoformans* and ste11 in *S. pombe*) [50–52]. We evaluated the homology of the upregulated HMG-box genes on closely related Mucoromycotina species and we identified that these three HMG-box genes are homologs of Mucoromycotina SexM HMG-box genes (Table 1).

**Table 1:** HMG-box genes significantly and specifically upregulated in the coinoculation treatment on host genotype COL2215.

Gene	Name	Homolog to	Blast hit Q. Cover/*E*-value/% Identity	Accession	Description
GBC53331.1	HMG-box	SexM/*M. mucedo*	31%/2e−08/28.70% Recip: 52%/3e−13/29.00%	AFA26123.1	SexM sex-determination transcription factor, contained in Mucoromycota MAT-locus [26]
GBC31594.1	HMG-box	minus sex/*R. delemar*	42%/1e−06/30.53% Recip: 53%/2e−12/30.53%	ADT91565.1	SexM sex-determination transcription factor, contained in Mucoromycota MAT-locus [26]
GBC37885.1	HMG-box	SexM/*S. megalocarpus*	40%/2e−10/37.11% Recip: 63%/7e−16/37.11%	AET35419.1	SexM sex-determination transcription factor, contained in Mucoromycota MAT-locus [26]

Blast hit to the nonredundant database targeting the Mucorales taxa. We show the reciprocal blast hit between the homolog sequences, the accession and description of each gene.

We reconstructed the phylogenetic relationships among the different HMG-box genes involved in sexual reproduction in fungi, including the three specifically upregulated HMG-box genes. We found that the three upregulated HMG-box genes were grouped in an independent clade within the HMG-box genes involved in fungal sexual reproduction (Supplementary Fig. 1). Finally, to verify whether the three upregulated HMG-box genes were homologs of the HMG-box genes encoded in the MAT-locus of Mucoromycotina species, we investigated the homologs of the three upregulated HMG-box genes in two different *M. circinelloides* and two different *Rhizopus oryzae* genome assemblies. Remarkably, we observed a single significant match of the HMG-box GBC53331.1 to the MAT-locus-encoded HMG-box gene in all the four genome assemblies (Supplementary File 7). We also observed a single significant match of the HMG-box GBC31594.1 to the MAT-locus-encoded HMG-box gene in three of the four genome assemblies. Finally, we did not observe any significant match of the HMG-box GBC37885.1 to the MAT-locus-encoded HMG-box genes on the four genome assemblies. These results suggest that HMG-box GBC53331.1 and GBC31594.1 are homologs of the MAT-locus-encoded HMG-box genes of Mucoromycotina species.

We further evaluated the composition of the loci containing the HMG-box gene homologs of encoded MAT-locus HMG-box (GBC53331.1 and GBC31594.1). We compared the synteny of HMG-box (GBC53331.1 and GBC31594.1) among published genome assemblies of genetically different *R. irregularis* isolates. We tested if the loci containing the HMG-box genes GBC53331.1 and GBC31594.1 displayed similarity to the MAT-locus of Mucoromycotina species (Supplementary Note 5). We found partial similarity between the *R. irregularis* locus containing HMG-box (GBC53333.1) and the MAT-locus of Mucoromycotina species. We observed that the region flanking the HMG-box (GBC53331.1) comprised a membrane transporter containing an EamA-like domain (thought to be the ancestor domain of the TPT family [53]), and the adapter protein coatomer gamma subunit (Sec21) (Supplementary Fig. 8a). In contrast, we did not find any similarity between flanking genes of locus containing HMG-box (GBC31594.1) and the MAT-locus of Mucoromycotina species. The HMG-box gene (GBC31594.1) was located in the second position of a locus containing a five-repeat tandem of HMG-box genes (Supplementary Fig. 8b). These results suggest that *R. irregularis* sex-determination could involve more than one locus containing HMG-box transcription factors.

### Specific upregulation of AMF genes in host genotype COL2215 that are homologs of genes involved in different stages of fungal mating in other fungal species

**Fig. 2:**
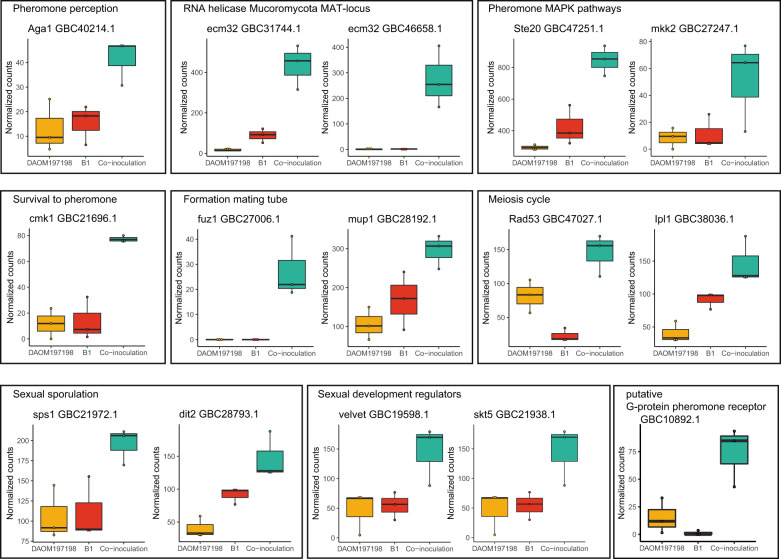
Differential transcription of *R. irregularis* genes in the roots of host genotype COL2215 that are known to be involved in different stages of mating responses in other fungi. The graphs show normalized counts of transcripts that displayed significantly higher gene transcription in the coinoculation treatment (with isolates B1 and DAOM197198 together) compared with the two single-inoculation treatments (B1 or DAOM197198). The number of genes reflects the genes that were found in common between (1) isolate DAOM197198 vs. coinoculation and (2) isolate B1 vs. coinoculation.

Out of the remaining 77 genes specifically upregulated in the coinoculation treatment compared with single-inoculation treatments, in host genotype COL2215, 15 of these were genes with a known function in different stages of mating in other fungal species (Fig. 2 and Table 2). These genes were homologs of genes involved in: (1) pheromone perception; (2) encoded in MAT-loci (HMG-box and RNA helicases); (3) involved in MAPK pathways (STE20 and Mkk2); (4) cell survival to pheromones; (5) formation of mating tubes; (6) meiosis; (7) sexual sporulation; and (8) mating regulators (see Supplementary Note 6 for a detailed description of the 15 upregulated genes).

**Table 2:** Fifteen additional genes that were significantly differentially transcribed between the two single-inoculation treatments and the coinoculation treatment in host genotype COL2215 and which are involved in recombination and sexual reproduction in other fungal species.

Gene	Name	Homolog to	Blast hit Q. Cover/*E*-value/% Identity	Accession	Description
GBC31744.1	Ecm32p	RNA helicase/*R. oryzae*	49%/1e−78/25.59% Recip: 56%/3e−12/19.71%	ADU02296.1	RNA helicase comprised in MAT-locus of Mucoromycota species [26].
GBC46658.1	Ecm32p	rna helicase/*L. corymbifera*	54%/3e−73/32.28% Recip: 73%/4e−77/32.54%	CDH53899.1	RNA helicase comprised in MAT-locus of Mucoromycota species [26].
GBC19598.1	Velvet factor	veA/*A. nidulans*	71%/6e−21/31.07% Recip: 67%/4e−23/31.00%	AAD42946.1	Positively regulates sexual development and negatively regulates asexual development [60].
GBC21938.1	Skt5p	SKT5/*S. cerevisiae*	54%/1e−21/30.49% Recip: 56%/2e−21/30.49%	CAA84882.1	The Skt5p homolog in *Schizosaccharomyces pombe* (27.586 % identity, 2.93e−14 *E*-value) is a regulatory gene that is involved in septum formation in fungi (Matsuo et al. 2004) and is required for chitin synthesis during mating in *Saccharomyces cerevisiae* [61].
GBC27006.1	Mynd domain protein	Fuz1/*U. maydis*	17%/3e−07/50.00% Recip: 8%/5e−07/50.00%	ABS85543.1	Necessary for the formation of the conjugation tube between opposite mating type isolates [62].
GBC21696.1	Cmk1p	CMK1/*S. cerevisiae*	47%/1e−17/31.96% Recip: 44%/1e−17/31.96%	KZV11616.1	In *S. cerevisiae*, exposure to pheromones induces sexual differentiation, but also induces cell death (Severin and Hyman 2002). CMK1 encodes a Ca2+/calmodulin-dependent protein kinase (Pausch et al. 1991) which is involved in the cell survival to pheromones [63].
GBC21972.1	Sps1p	SPS1/*S. cerevisiae*	40%/4e−20/25.48% Recip: 56%/4e−20/25.48%	KZV12764.1	In *S. cerevisiae*, SPS1 is transcribed at the end of meiosis and plays a role in the spore wall development [64].
GBC27247.1	Mkk2p	MKK2/*S. cerevisiae*	30%/1e−19/30.92% Recip: 30%/2e−19/30.92%	KZV07375.1	MKK2 is involved in the cell wall integrity MAPK kinases cascade, which is associated to the pheromone response during mating in budding yeast [32].
GBC28192.1	Mup1p	MUP1/*S. cerevisIae*	87%/6e−32/24.56% Recip: 90%/6e−32/24.56%	KZV11281.1	MUP1 is a methionine transporter. In *U. maydis* methionine auxotrophs lack the ability to from mating tubes, but this defect is restored with the addition of methionine [65].
GBC28793.1	Dit2p	DIT2/*S. cerevisiae*	97%/3e−30/26.02% Recip: 95%/5e−30/25.61%	KZV12646.1	Dit2 is a sporulation-specific gene, which in yeast, is involved in the formation of the surface layer of ascospores [66].
GBC38036.1	Ipl1p	IPL1/*S. cerevisiae*	34%/6e−20/27.52% Recip: 68%/2e−20/25.61%	KZV07303.1	In yeast, IPL1 is involved in segregation cycles during meiosis [67].
GBC10892.1	Aig1	GPA1/*S. cerevisiae*	11%/0.11/32.43%^a^ Recip: 15%/0.11/32.43%^a^	KZV10725.1	Guanine nucleotide-binding G-protein which is part of the pheromone receptor [68].
GBC40214.1	Tpk1p	Aga1/*U. maydis*	74%/8e−22/23.14% Recip: 42%/2e−20/25.28%	XM_751538.1	Aga1 is a cAMP-dependent protein kinase, which in *U. maydis* is involved in pheromone perception [69].
GBC47027.1	Rad53p	Rad53/*S. cereviseae*	34%/4e−21/27.27% Recip: 41%/3e−21/27.27%	KZV07361.1	RAD53 have an important role in maintaining genomic integrity after DNA damage during mitosis [70] and during meiosis [71].
GBC47251.1	Ste20p	STE20/*S. cereviseae*	67%/4e−17/26.57% Recip: 38%/7e−17/26.57%	KZV10712.1	STE20 is involved in several MAPK kinases cascades associated to the pheromone response during mating in budding yeast [32].

^a^No significant blast hit, however, gpa1 and aga1 share the P-loop_NTPase super family containing the guanine nucleotide-binding region.

Together, these results suggest that different stages of a putative mating response were elicited when two genetically different *R. irregularis* isolates coexisted *in planta* in the host plant genotype COL2215.

### No upregulation of putative MAT-locus homeodomain genes

A previous study identified a putative MAT-locus in *R. irregularis* that is similar to the MAT-locus of basidiomycete fungi, containing two HD genes organized in opposite transcriptional direction [8]. However, no evidence has ever been presented demonstrating that HD transcription factors are only transcribed in the presence of two genetically different *R. irregularis* isolates. We identified the homologs of HD2 and HD1-like genes in the gene annotation from this study (Supplementary Fig. 9a, b) and looked at their transcription in the single and coinoculation treatments on the three different host plant genotypes. We did not find any reads mapping to HD2 (Supplementary Fig. 9c) and we did not observe any significant difference in gene transcription between the treatments for HD1-like (Supplementary Fig. 9d).

### Host-differential activation of genes involved in the mating response

**Fig. 3:**
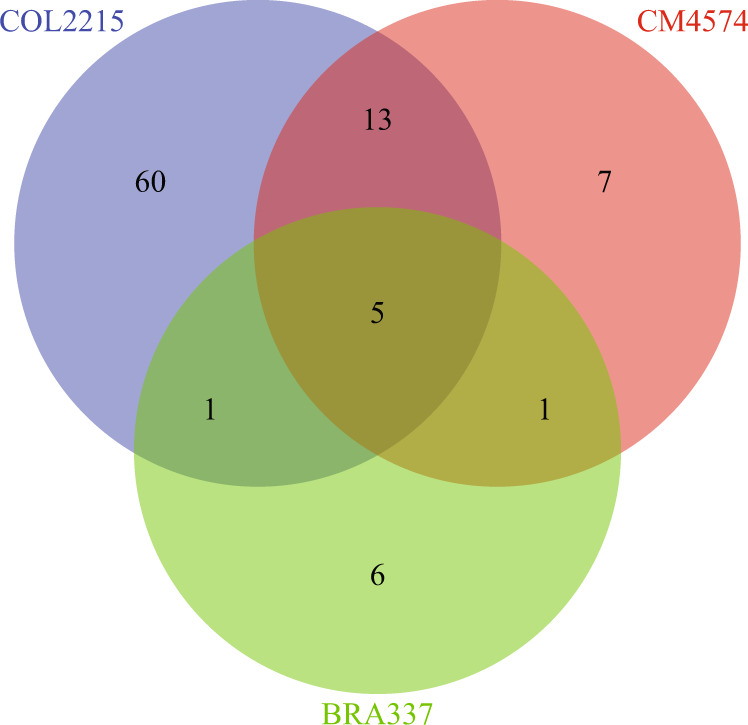
Venn diagram representing number of genes differentially transcribed between both single-inoculations and the coinoculation treatment on the three different host genotypes. Each number reflects the genes that were found in common between (1) isolate DAOM197198 vs. coinoculation and (2) isolate B1 vs. coinoculation for each host plant genotype.

We identified different upregulated genes in the coinoculation treatment compared with both single-inoculation treatments among the three plant genotypes (COL2215: 79 genes, CM4574: 26 genes, and BRA337: 13 genes; Fig. 3). We observed that compared with host genotype COL2215, fewer genes were specifically upregulated in the coinoculation treatments in host genotypes CM4574 and BRA337.

We observed that 26 genes were specifically upregulated in the coinoculation treatment with host genotype CM4574 (Supplementary File 8). Of these, 18 genes were differentially transcribed in the coinoculation treatment in both host genotypes CM4574 and COL2215. These genes included several that are known to be involved in mating and sexual reproduction in other fungi: the GBC53331.1 HMG-box gene, the RNA helicase ecm32 (GBC31744.1), the MAPK STE20 gene (GBC47251.1), the sexual development regulators veA (GBC19598.1) and skt5 (GBC21938.1), the gene cmk1 (GBC21696.1) which is involved in the survival to pheromone exposure, the dit2 gene (GBC28793.1) which is involved in sexual sporulation, and the G-protein Aig1 (GBC10892.1).

The interaction between the two isolates in symbiosis with host genotype BRA337 resulted in the upregulation of 13 genes that were differentially transcribed between the coinoculation treatment compared with the two single-inoculations (Supplementary File 9). From this set we identified five genes that were also differentially transcribed in symbiosis with host genotype COL2215 and host genotype CM4574; namely, HMG-box: GBC53331.1, the RNA helicase: GBC31744.1, the two sexual development regulators; veA: GBC19598.1 and skt5: GBC21938.1 and the G-protein Aig1: GBC10892.1.

**Fig. 4:**
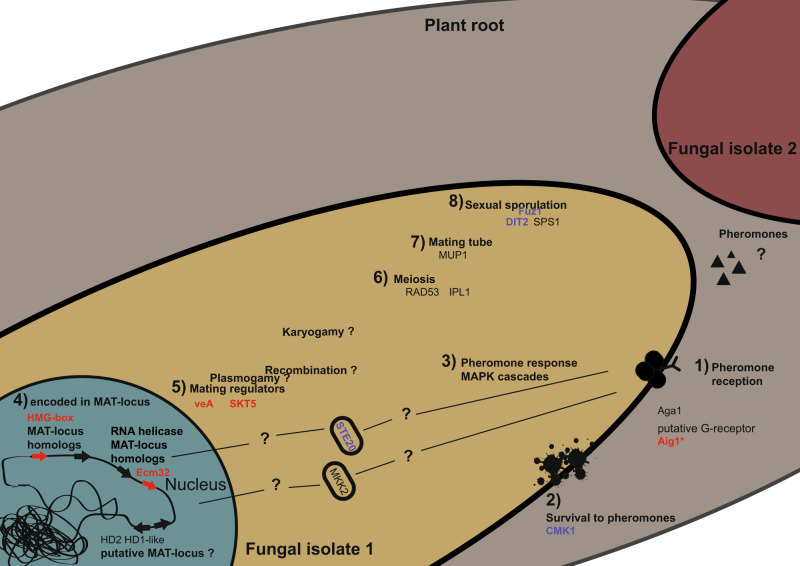
Schematic representation of the putative mating response in *R. irregularis*. We identified homologs of genes involved in (1) pheromone reception, (2) survival to pheromones, (3) different pheromone response MAPK cascades, (4) encoded in MAT-locus, (5) mating regulation, (6) meiosis, (7) formation of the mating tube, and (8) sexual sporulation. We did not observe upregulation of homeodomains proposed on a putative *R. irregularis* MAT-locus. We did not find upregulation of genes encoding the mating pheromones or those involved in plasmogamy, karyogamy, and recombination. Genes in blue were observed in host plant genotypes COL2215 and CM4574. Genes in red were observed in the three host plant genotypes in the coinoculation treatment specifically.

In summary, all the genes related to a putative fungal mating responses identified in host genotypes CM4574 and BRA337 were detected in host genotype COL2215. The results from the three host genotypes taken together represent the first demonstration of genes involved in several steps of a putative mating response in AMF (Fig. 4).

Few studies have evaluated genome-wide gene transcription during the mating response in Mucoromycota species. In order to understand if the list and number of activated genes obtained in this study was representative of the genes expected to be identified in coinoculation studies compared with single-inoculations, we compared the results found in this study to an experiment that compared gene transcription in a coinoculation treatment to a single-inoculation treatment in the Mucoromycotina species *Rhizopus microsporus* [54]. In that experiment, the authors identified a set of genes transcribed during confirmed mating in *R. microsporus* from a list of 99 genes known to be involved in sexual reproduction in diverse fungal taxa. We evaluated the homology of the coinoculation-specific differentially transcribed genes in *R. irregularis*, to the gene set of 99 mating and reproduction genes across fungi (Supplementary File 10). In *R. irregularis*, we observed that similar a fraction of the total gene set was upregulated at a given time to that observed during sexual reproduction between two different *R. microsporus* isolates (Supplementary Fig. 10). Some of these genes were common to both organisms but a number of genes were either activated in *R. irregularis* but not in *R. microsporus* or *vice versa*.

### Different levels of coexistence in the coinoculation samples revealed by SNP analysis of RNA-seq data

**Fig. 5:**
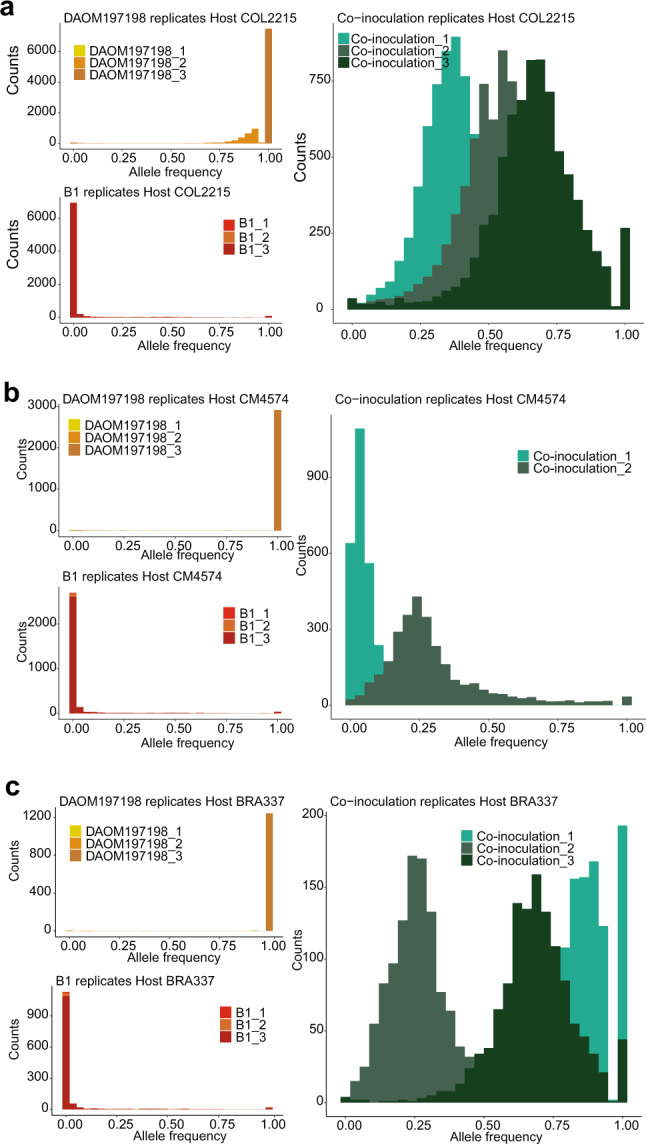
Identification of the proportion of each isolate in each sample. Allele frequency distributions for each sample in host genotype: **a** COL2215, **b** CM4574, and **c** BRA337. We plotted the allele frequency distribution of the reference and alternative allele in the single-inoculation and the coinoculation samples. Please note that single-inoculations display a single peak either at 100% reference allele or 100% alternative allele. In contrast, the coinoculation samples display different proportions of each isolate represented by the peak of the distribution.

We analyzed SNPs in the *R. irregularis* RNA-seq data set in order to confirm the presence of the two isolates within the coinoculation samples. We identified positions with no missing data across the samples, where isolate DAOM197198 displayed the reference allele (previously samples had been mapped to isolate DAOM197198) and where isolate B1 displayed an alternative allele. In total, we kept 7656 positions from transcripts found in plant genotype COL2215, 1255 positions in plant genotype BRA337, and 2945 positions in plant genotype CM4574 (Supplementary File 11). We observed for almost all the positions, independently of the host plant genotype, that isolate DAOM197198 displayed the reference allele, isolate B1 displayed an alternative allele and the coinoculation samples displayed a combination of both reference and alternative alleles (see Supplementary Fig. 11, for an example of three random positions in each cultivar). We then combined all the positions together to make allele frequency distributions of each sample. We observed that the majority of positions in isolate DAOM19798 displayed an allele frequency of 1, meaning that the majority of positions displayed the reference allele. In contrast, the majority of positions in isolate B1 displayed an allele frequency of 0, meaning that the majority of positions displayed the alternative allele. Finally, we observed that the coinoculation samples displayed intermediate levels of allele frequency confirming that both isolates indeed coexisted in the coinoculation treatment (Fig. 5). We observed that in plant genotype COL2215 the three samples displayed the ratios 41:59, 55:45, and 66:34 of isolate DAOM197198 to B1, respectively (Fig. 5a). In contrast, in plant genotype CM4574, isolate B1 predominantly colonized the plant roots in the ratio 8:92 in one sample and 30:70 in the second sample of isolate DAOM197198 to B1, respectively (Fig. 5b). In plant genotype BRA337, there was as also an uneven coexistence of both isolates with a ratio of 82:18, 30:70 and 68:32 of isolate DAOM197198 to B1 (Fig. 5c). Thus, we observed a more even coexistence in host genotype COL2215, where the greatest number of specifically upregulated genes where detected.

## Discussion

We used an experimental approach to identify AM fungal genes that were exclusively transcribed *in planta* when two genetically different *R. irregularis* isolates coexisted in the same roots. We did not observe any concordant plant growth response to the coinoculation treatments. However, we observed several fungal genes that were differentially transcribed in the coinoculation treatments compared with the single-inoculations. Some of these genes specifically upregulated on the coinoculation treatments were known to be involved in different steps of mating or sexual reproduction in other fungi organisms. This represents the only known AMF genes that are specifically upregulated when two genetically different isolates of the same AMF species coexist *in planta*. First, we identified two SexM HMG-box genes and two RNA helicases that were homologs of genes encoded in the MAT-locus of Mucoromycotina species. Second, we observed specific upregulation of several genes involved in different steps of the fungal mating response (pheromone receptors, MAPK cascades, formation of conjugation tube, meiosis, and sexual sporulation). Third, we observed that differences in the number of upregulated genes found among different host plant genotypes could be explained by the coexistence of the two fungi inside roots. The upregulation of these genes in the coinoculation treatment compared with the single-inoculations suggests that different parts of the molecular machinery of a putative mating response could be elicited by AMF.

### Putative mating response between two genetically different *R. irregularis* isolates

A limitation of this study is that the functions of the upregulated genes were obtained based on homology and sequence comparison to other fungal model species. An appropriate approach to identify gene function in AMF would be to use reverse genetics methods such as host-induced gene silencing [55]. However, because of the heavy amount of work to develop these reverse genetics approaches, homology comparison is a good compromise to predict the function of a large number of genes.

The genes induced in coexistence in this study are homologs of genes involved in sexual reproduction in different fungal taxa. The comparison of this gene set to *R. microsporus* revealed that the majority of these genes did not overlap between *R. microspores* and *R. irregularis*. However, a similar number of genes of this gene set were expressed in *R. irregularis* compared with a fungal species known to be undergoing mating. However, differences in the activation of genes involved in sexual reproduction between species of different taxa are common, as different taxon groups can share and have different genes related to sexual reproduction [54]. In consequence, it is not surprising that few genes induced in coexistence were shared between these two species from different phyla.

The results observed in the three host plant genotypes suggest that the mating response was further developed in host genotype COL2215 compared with the two other host plant genotypes. In addition, it highlights a high conservation of the activation of five genes that were upregulated in the coinoculation treatment compared with the single-inoculation treatment in all three hosts: the HMG-box (GBC53331.1) and the RNA helicase (GBC31744.1), the homologs of which are found in the MAT-locus of Mucoromycotina species, the two sexual development regulators (velvet and skt5) and the G-protein Aig1 (GBC10892.1). These latter five genes could be used as genetic markers of a putative mating response between AMF isolates.

In this experiment, we were interested in the gene transcription response of nonself-interactions between two isolates of the same species coexisting inside the plant, without specifically looking for mating. Remarkably, we were able to identify genes involved in different steps of a putative mating comprising pheromone reception, MAPK kinases, encoded on an MAT-locus, mating regulators, involved in formation of the mating tube, meiosis, and in sexual sporulation. Although, the discoveries in this experiment suggest that different steps of a putative mating response were activated when two different isolates coexist, the experimental design used in this experiment is not suited to demonstrate that sexual reproduction is happening. A final demonstration of sexual reproduction in AMF should include the identification of recombinant progeny after coexistence of two different isolates of the same AMF species.

### Does *R. irregularis* possess a single MAT-locus containing a homeodomain gene or a multilocus sex-determining region containing HMG-box genes?

We identified two upregulated HMG-box genes in the coinoculation treatment compared with the single-inoculations that were homologs of the HMG-box encoded in the MAT-locus of Mucoromycotina species but we did not observe upregulation of HD genes encoded in the putative MAT-locus previously identified.

Basidiomycetes display a loss of the HMG-box and a gain of HDs in their MAT-loci. This is in contrast to species from the Mucoromycotina subphylum, where sex-determination is regulated by HMG-box transcription factors [29]. The existence of an HD as a sex-determination transcription factor in *R. irregularis* challenges the hypothesis that an HMG-box transcription factor is the ancestral sex-determinant in the fungal kingdom [29, 56]. Two hypotheses could emerge from the results concerning sex-determination genes. First, it could be possible that the HD genes are not involved in mating and that HMG-box genes are involved in the mating when two genetically different individuals encounter each other. An alternative hypothesis is that HD genes and HMG-box genes are both required for mating, but their induction occurs at different times following the meeting of two fungi. The lack of any evidence concerning transcription of HD genes located in the putative MAT-locus (described by Ropars et al. [8]) when two *R. irregularis* isolates coexist, means that a deeper evaluation of the temporal dynamics of both HMG-box and HD genes is needed in order to confirm whether *R. irregularis* display a single MAT-locus containing HD genes or if it displays a multilocus architecture also containing HMG-box genes.

### Do mating responses only occur *in planta*?

A unique feature of this study, compared with other studies on transcription of AM fungal genes involved in mating and sexual reproduction, is that we successfully detected the upregulation of genes that only occurred when two genetically different *R. irregularis* isolates coexisted. Most notably, we identified the activation of genes when the fungi coexist *in planta.* Previous published studies focussed on transcriptional responses occurring when extra-radical hyphae of the two fungal individuals met in an in vitro culture system growing on a sterile artificial medium [36].

Although we did not test whether the activation of a putative mating response is only present *in planta*, it is possible that this mechanism also occurs in extra-radical mycelia. Anastomosis between different isolates of the same species has been shown to be rare, but more likely when the isolates are genetically similar than when they are genetically distant [11]. The two *R. irregularis* isolates used in this study (DAOM197198 and B1) belong to different genetic groups and from different locations [57]. These isolates have never been tested for extra-radical mycelia anastomosis, so it is possible that anastomosis could occur between these two isolates. Studies of coexistence between extra-radical mycelia of different AMF genotypes are still needed to better understand the nature of the putative mating response in *R. irregularis*.

### Host-differential putative mating response in *R. irregularis*

In this experiment, we observed that the number of differentially transcribed genes in the coinoculation treatment was altered by host plant genotype. When we looked at the presence of each isolate in the coinoculation treatment, we observed that coexistence was more even in host genotype COL2215. In contrast, coexistence between the isolates was less evenly distributed (towards dominance by one isolate) in host genotypes CM4574 and BRA337. It is likely that the activation of genes involved in a putative mating response could be influenced by the proportion of both coexisting fungi, where a more even distribution would maximize the signal and a less even distribution would decrease the signal. Thus, we suspect that the small number of genes detected in host genotype CM4574 and BRA337 were the result of an uneven colonization of both isolates.

Several hypotheses could explain uneven colonization of the different isolates in the coinoculation samples. First, it is possible that direct fungal competition or plant mediated competition could be responsible of the proportion of each isolate in the coinoculations [6]. It was previously experimentally demonstrated that both AM fungal and plant genetic re-programming in the symbiosis is strongly affected by the plant genotype [37]. Thus, the host genotype could influence faster growth of one of the fungal isolates, thus, giving rise to more uneven colonization of the two fungi than in other host genotypes. An alternative hypothesis to explain the differences regarding which fungal genes were specifically upregulated in coinoculation treatments among host genotypes is that the host plant influences the recognition and subsequent mating between two isolates by providing the required conditions for the fungus. Evidence of a role of host plant derivatives on fungal traits was recently observed in *R. irregularis*, where asexual sporulation was promoted by abscisic acid and other root volatiles [58]. In conclusion, further research should be focussed on determining if the host plant has an “indirect” or “direct” effect on the putative mating response in *R. irregularis*.

Population genetics studies of *R. irregularis* indicate that sexual reproduction in these fungi is probably very rare [16] and this makes detection of sex in these fungi highly elusive to experimenters. This study experimentally demonstrates that two different isolates of *R. irregularis* can recognize each other *in planta* and could elicit a putative mating response, involving a large majority of molecular mechanisms already known in other fungal species. In consequence, this study represents an important step in the identification of the molecular mechanisms of recognition and mating in AMF. Our discovery of *in planta* activation of genes related to different stages of mating in *R. irregularis* also provides some clues to understanding the early steps of the evolution of sex-determination of fungal systems. AMF are enormously important for plant growth and comprehending sex in these fungi is key for generating genetically diverse isolates having differential effects on plant growth that could lead to their more directed use in agriculture [59].

## Supplementary information

Supplementary legends

Supplementary figures and notes

Supplementary file 1

Supplementary file 2

Supplementary file 3

Supplementary file 4

Supplementary file 5

Supplementary file 6

Supplementary file 7

Supplementary file 8

Supplementary file 9

Supplementary file 10

Supplementary file 11
