# Supplementary material for: Coexistence of genetically different Rhizophagus irregularis isolates induces genes involved in a putative fungal mating response
Source: ISME J. 2020 Jun 8;14(10):2381–94. doi: 10.1038/s41396-020-0694-3 (PMC7490403; doi:10.1038/s41396-020-0694-3)
Supplement: Supplementary file 1 — Supplementary legends [file 41396_2020_694_MOESM1_ESM.docx]

**Co-existence of genetically different *Rhizophagus irregularis* isolates induces genes involved in a putative fungal mating response**

Ivan D. Mateus*, Edward C. Rojas, Romain Savary, Cindy Dupuis, Frédéric G. Masclaux, Consolée Aletti & Ian R. Sanders*

**Supplementary legends**

**Supplementary Fig. 1. Schematic representation of the experimental design.** **a**

Representation of the experiment comprising four treatments: Single-inoculation with isolate B1, single-inoculation with isolate DAOM197198, co-inoculation with isolates B1 and DAOM197198 and mock-inoculated. All treatments were performed with three different cassava cultivars (genotypes). **b** RNA sequencing and bioinformatic analysis workflow. RNA extracted from fine roots was sequenced with Illumina Hi-seq 2500 paired-end sequencing. Sequences were aligned them to the cassava genome assembly. Remaining unmapped reads were retained and then mapped to the AM fungal genome assembly, producing a dataset comprising only AM fungal reads. **c** Schematic representation of the expected transcription profile of a gene involved exclusively in the interaction between the two AMF isolates.

**Supplementary Fig. 2. RNA-seq quality.** Comparison of the total number of reads, number of reads mapped to the cassava genome, number of reads mapped to the *R. irregularis* genome and number of fungal features with more than 5 counts for each treatment. Data is shown for **a** host plant genotype COL2215, **b** host plant genotype CM4574 and **c** host plant genotype BRA337. In (*) are shown the statistically significant differences between pairs of treatments.

**Supplementary Fig. 3. Principal component analysis of normalized gene counts from *R. irregularis* transcripts in the inoculation treatments in 3 cassava genotypes.** **a** COL2215, **b** CM4574 and **c** BRA337.

**Supplementary Fig. 4. Heatmap of sequence containment in raw data.** The reference genome assemblies are represented in rows. The samples are represented in the columns. The samples were clustered by row and column values. Shared hashes represent the amount of each sequence in a given sample.

**Supplementary Fig. 5. Fungal colonization detected by the grid-intersect method in each treatment.** The Y axis describes the fungal colonization as a percentage of intersects with fungal structures detected / total number of intersects. Data is shown independently for **a** host plant genotype COL2215, **b** host plant genotype CM4574 and **c** host plant genotype BRA337. * denotes a statistically significant difference between a given pair of treatments.

**Supplementary Fig. 6. Plant growth responses.** We show the total dry weight, belowground dry weight and aboveground dry weight of the different treatments of **a** host plant genotype COL2215, **b** host plant genotype CM4574 and **c** host plant genotype BRA337. * denotes a statistically significant difference between a given pair of treatments. The Y axis represent the weight in grams.

**Supplementary Fig. 7. Phylogenetic reconstruction of the HMG-boy domain and alignment of HMG-box genes in fungi. a** Maximum likelihood phylogenetic reconstruction of the HMG-box domain of HMG-box genes involved in sexual reproduction in fungi, including the three upregulated HMG-box genes observed in this study (shown in bold type). LG + G was selected as the substitution model and we performed 100 bootstraps. We show the accession numbers of the different HMG-box genes. **b** Alignment of the conserved domain of HMG-box genes involved in sexual reproduction in fungi including the three upregulated HMG-box genes observed in this study (shown in bold type).

**Supplementary Fig. 8. Synteny plot of the contigs containing the induced HMG-box genes**. **a** GBC53331.1 and **b** GBC31594.1 in five genetically different *R. irregularis* isolates DAOM197198, A1, A5, A4 and C2. Grey lines linking the strains represent homologous regions among the isolates. The induced HMG-box gene is represented in green. *The fifth HMG-box gene in isolates DAOM197198 and C2 on the locus containing GBC31594.1 was not detected as an HMG-box domain because of an amino acid substitution in the N-terminal part of the sequence and is, therefore, labelled “hypothetical protein”.

**Supplementary Fig. 9. Analysis of transcription of homeodomain HD2 and HD1-like genes encoded in the putative MAT-locus proposed by Ropars *et al.,* 2016 in three genetically different cassava cultivars (COL2215, CM4574 and BRA337).** **a** Blast homology of HD2 and HD1-like genes to the gene-prediction used in this study. **b** Gene organization of the putative MAT-locus in *R. irregularis* DAOM197198. **c** Gene transcription of HD2 and **d** HD1-like genes in single inoculations and the co-inoculation treatments of this study. We show the normalized counts per treatment.

**Supplementary Fig. 10. Genes potentially involved in sexual reproduction in *R. irregularis*.** Homologs to reproductive proteins across a fungal gene set defined by Mondo *et al.,* 2018. We highlighted all blast hits to the reproductive proteins in this fungal gene list of *R. irregularis* genes that were significantly differentially transcribed in the co-inoculation treatment compared to the single-inoculations in host genotypes COL2215 (blue), CM4574 (green) and BRA337 (brown). As a comparison we show the genes that were differentially transcribed during confirmed sexual reproduction in the Mucoromycotina species *Rhizopus microsporus* [1] (grey). For comparative purposes, genes names were obtained and shown as in Mondo *et al.,* 2017. Consequently, gene names differ from the gene names described in this study.

**Supplementary Fig. 11. Analysis of SNPs in RNA-seq data of three random individual positions.** Allele counts of the reference or alternative allele in each sample. **a** data from host plant genotype COL2215, **b** host plant genotype CM4574 and **c** host plant genotype BRA337.

**Supplementary file 1**. Source code describing the steps of the bioinformatic analysis and differential gene-transcription analysis

**Supplementary file 2.** Summary of RNA sequencing results per sample and associated statistics.

**Supplementary file 3**. Identification of fungal species sequences in raw sequencing files. Output from Mash screen --winner-take-all strategy function. The mash screen analysis included 1721 fungal genomes available in NCBI

**Supplementary file 4**. Detail on fungal colonisation and associated statistics.

**Supplementary file 5**. Plant growth responses and associated statistics.

**Supplementary file 6**. Differential transcription analysis, normalized gene counts and gene annotation. We show the genes significantly differentially transcribed between the co-inoculation treatment and isolate B1 and the co-inoculation treatment and isolate DAOM197198 on host genotype COL2215.

**Supplementary file 7**. Upregulated HMG-box genes display homologs on Mucoromycota genome assemblies. a) Genome assemblies tested for HMG-box homology. b) Blast hit table of upregulated HMG-box genes on the different genome assemblies. In bold the significant blast hits. *Different from HMG-box encodet MAT-locus c) Conserved domains found in the region neighbouring the significant blast hit.

**Supplementary file 8**. Differential transcription analysis, normalized gene counts and gene annotation. We show the genes significantly differentially transcribed between the co-inoculation treatment and isolate B1 and the co-inoculation treatment and isolate DAOM197198 on host genotype CM4574.

**Supplementary file 9**. Differential transcription analysis, normalized gene counts and gene annotation. We show the genes significantly differentially transcribed between the co-inoculation treatment and isolate B1 and the co-inoculation treatment and isolate DAOM197198 on host genotype BRA337.

**Supplementary file 10**. Blast hit results of differentially transcribed genes in the co-inoculation treatment compared to the single-inoculations on the three host genotypes to the gene list of reproductive proteins across fungi (Mondo et al., 2017)

**Supplementary file 11**. SNP calling from the VCF files in each of the three cassava varieties.
