# Supplementary material for: Coexistence of genetically different Rhizophagus irregularis isolates induces genes involved in a putative fungal mating response
Source: ISME J. 2020 Jun 8;14(10):2381–94. doi: 10.1038/s41396-020-0694-3 (PMC7490403; doi:10.1038/s41396-020-0694-3)
Supplement: Supplementary file 2 — Supplementary figures and notes [file 41396_2020_694_MOESM2_ESM.docx]

*Supplementary Figures*

**Co-existence of genetically different *Rhizophagus irregularis* isolates induces genes involved in a putative fungal mating response**

Ivan D. Mateus*, Edward C. Rojas, Romain Savary, Cindy Dupuis, Frédéric G. Masclaux, Consolée Aletti & Ian R. Sanders*

**
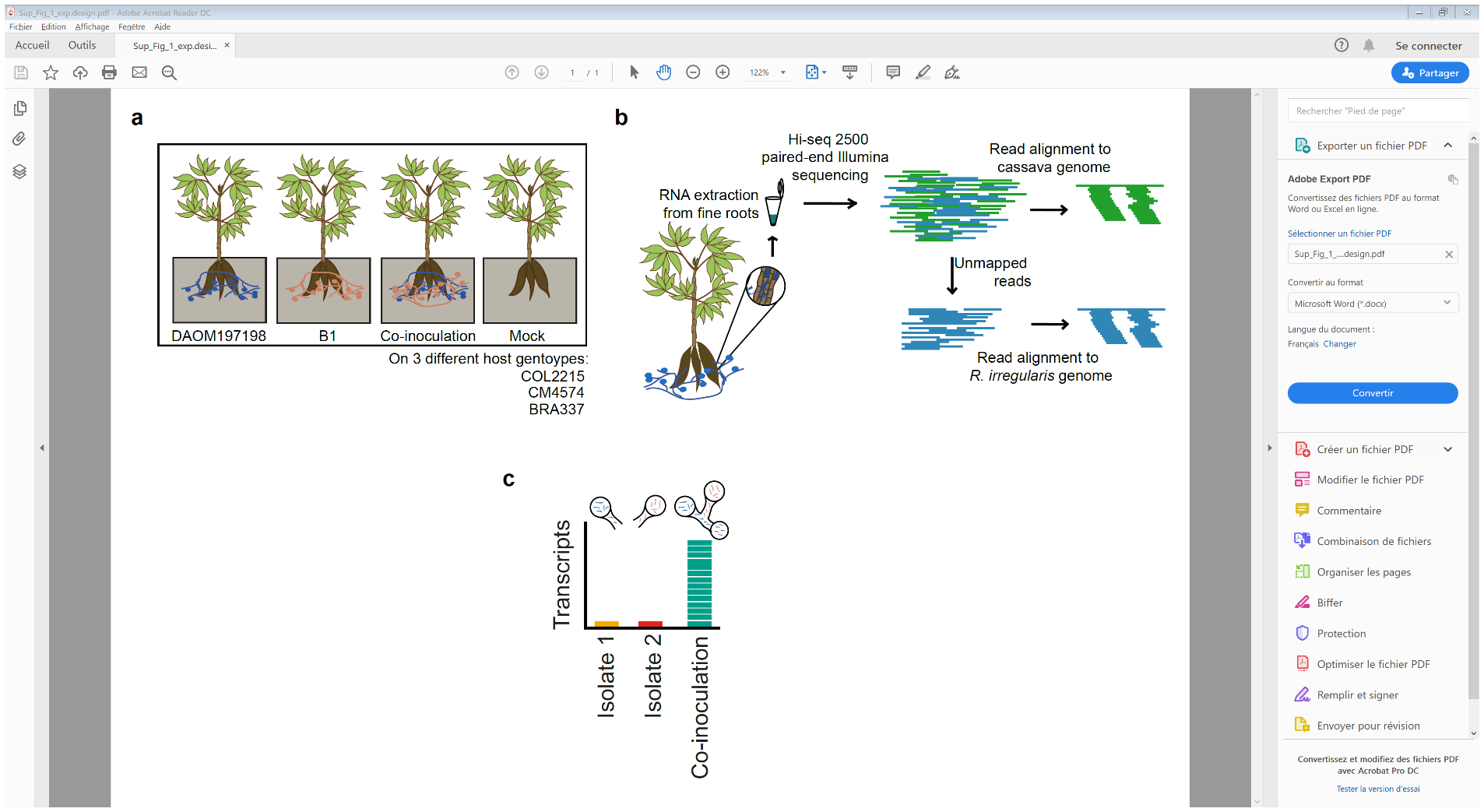
**

**Supplementary Fig. 1. Schematic representation of the experimental design.** **a**

Representation of the experiment comprising four treatments: Single-inoculation with isolate B1, single-inoculation with isolate DAOM197198, co-inoculation with isolates B1 and DAOM197198 and mock-inoculated. All treatments were performed with three different cassava cultivars (genotypes). **b** RNA sequencing and bioinformatic analysis workflow. RNA extracted from fine roots was sequenced with Illumina Hi-seq 2500 paired-end sequencing. Sequences were aligned them to the cassava genome assembly. Remaining unmapped reads were retained and then mapped to the AM fungal genome assembly, producing a dataset comprising only AM fungal reads. **c** Schematic representation of the expected transcription profile of a gene involved exclusively in the interaction between the two AMF isolates.

**
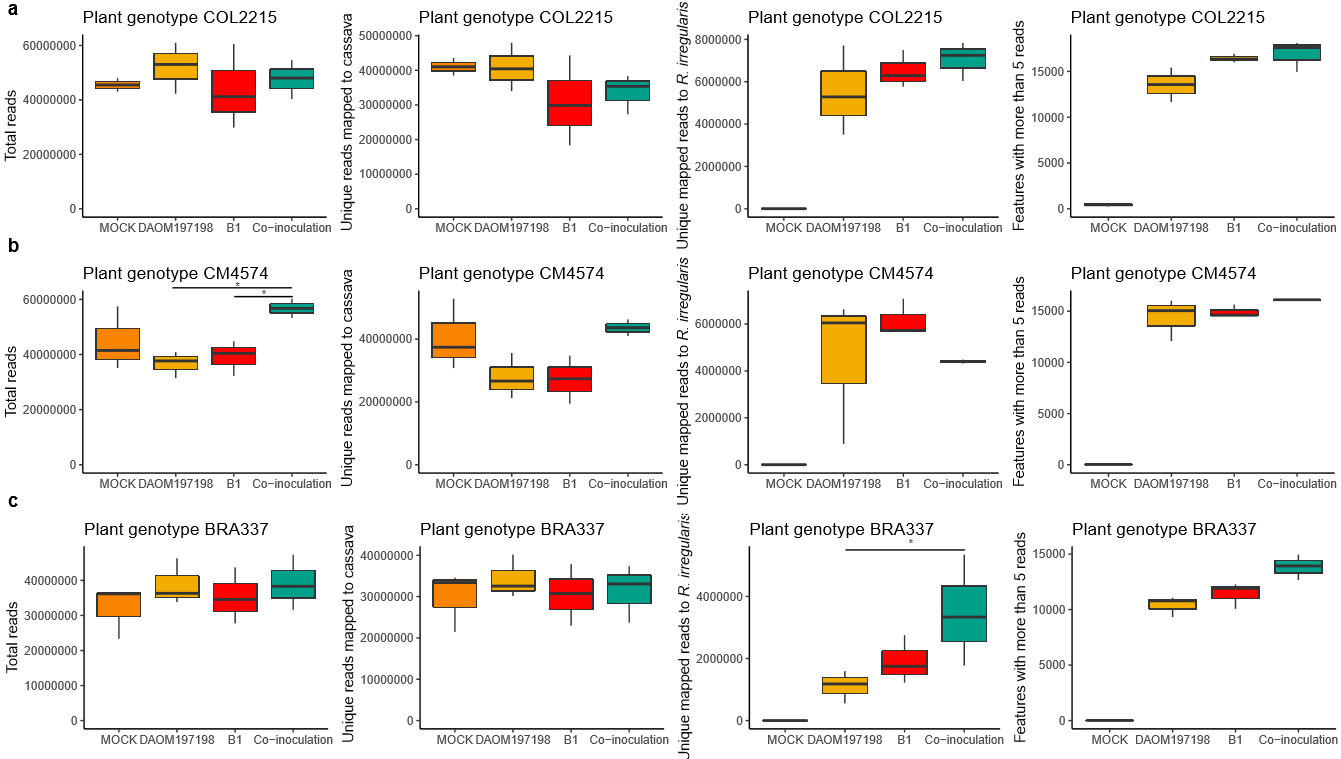
**

**Supplementary Fig. 2. RNA-seq quality.** Comparison of the total number of reads, number of reads mapped to the cassava genome, number of reads mapped to the *R. irregularis* genome and number of fungal features with more than 5 counts for each treatment. Data is shown for **a** host plant genotype COL2215, **b** host plant genotype CM4574 and **c** host plant genotype BRA337. In (*) are shown the statistically significant differences between pairs of treatments.


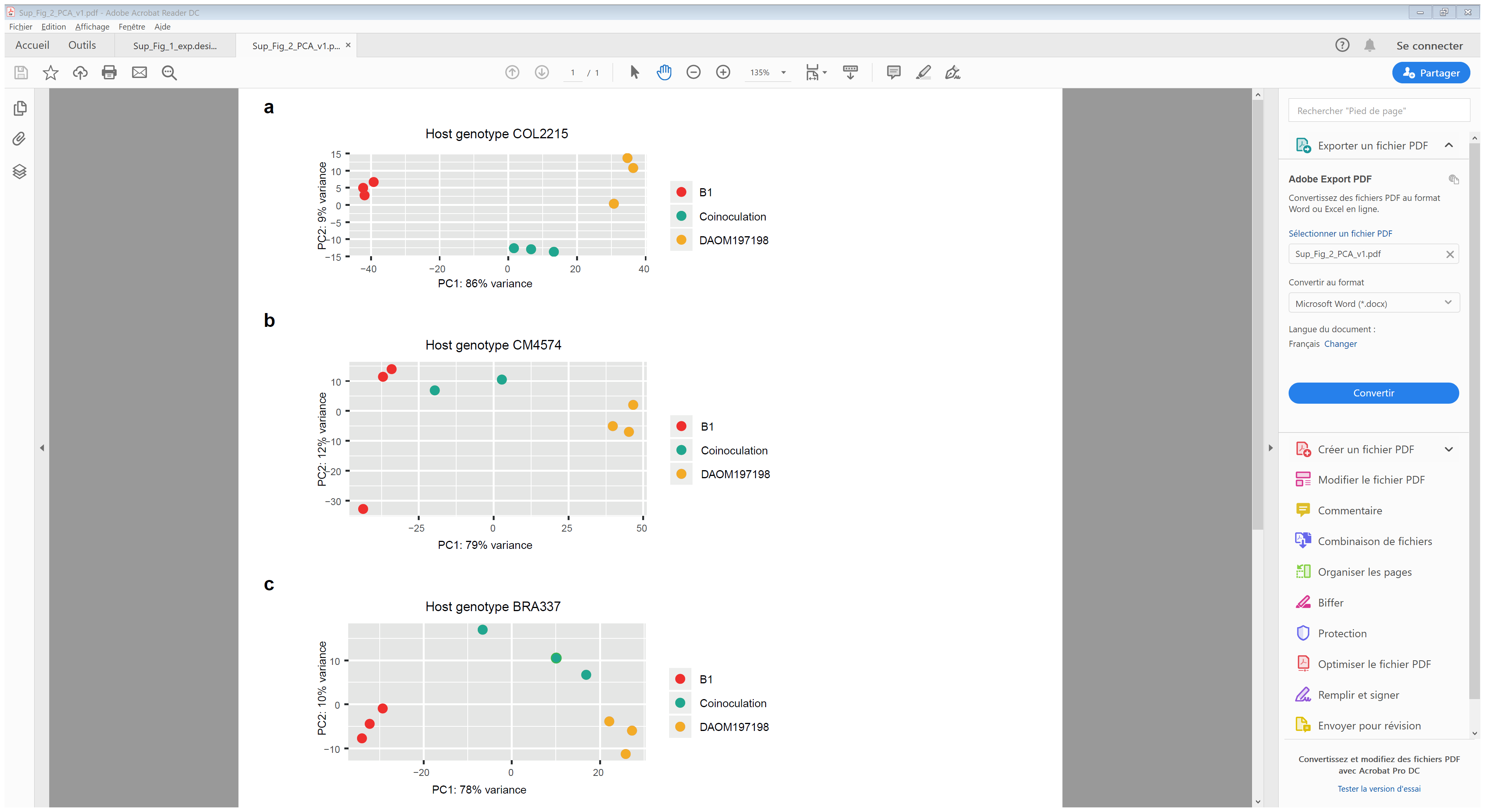


**Supplementary Fig. 3. Principal component analysis of normalized gene counts from *R. irregularis* transcripts in the inoculation treatments in 3 cassava genotypes.** **a** COL2215, **b** CM4574 and **c** BRA337.

**
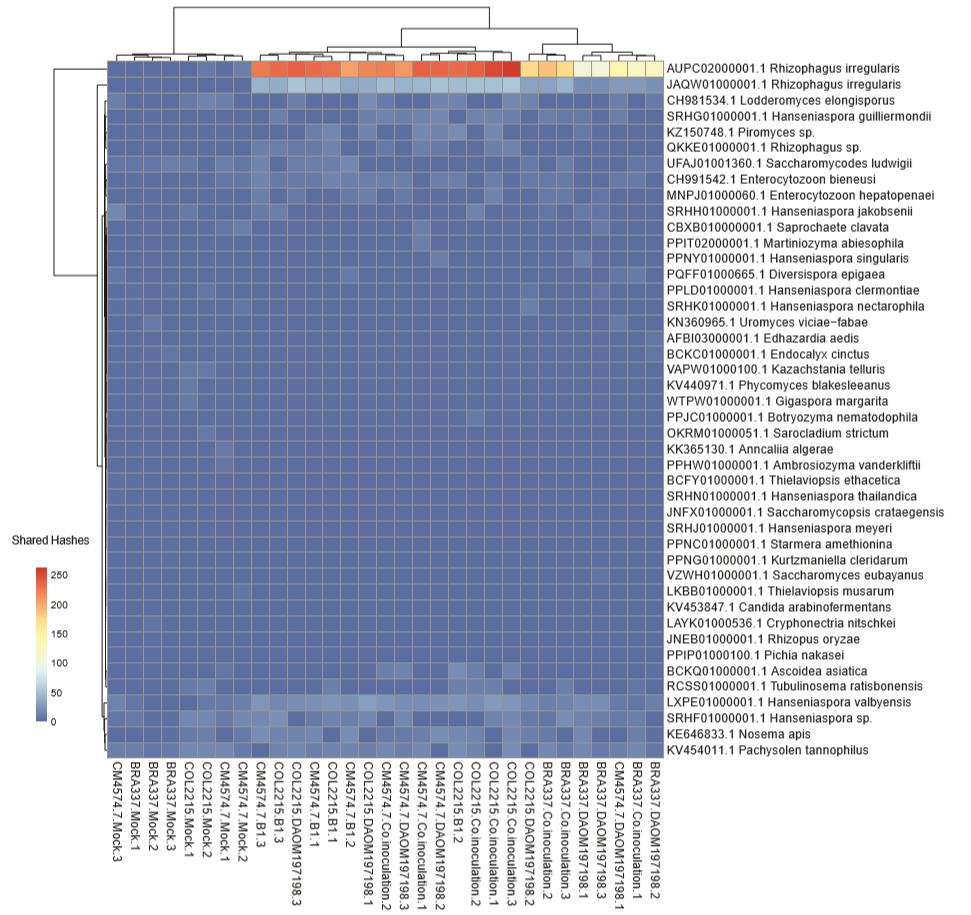
**

**Supplementary Fig. 4. Heatmap of sequence containment in raw data.** The reference genome assemblies are represented in rows. The samples are represented in the columns. The samples were clustered by row and column values. Shared hashes represent the amount of each sequence in a given sample.

**
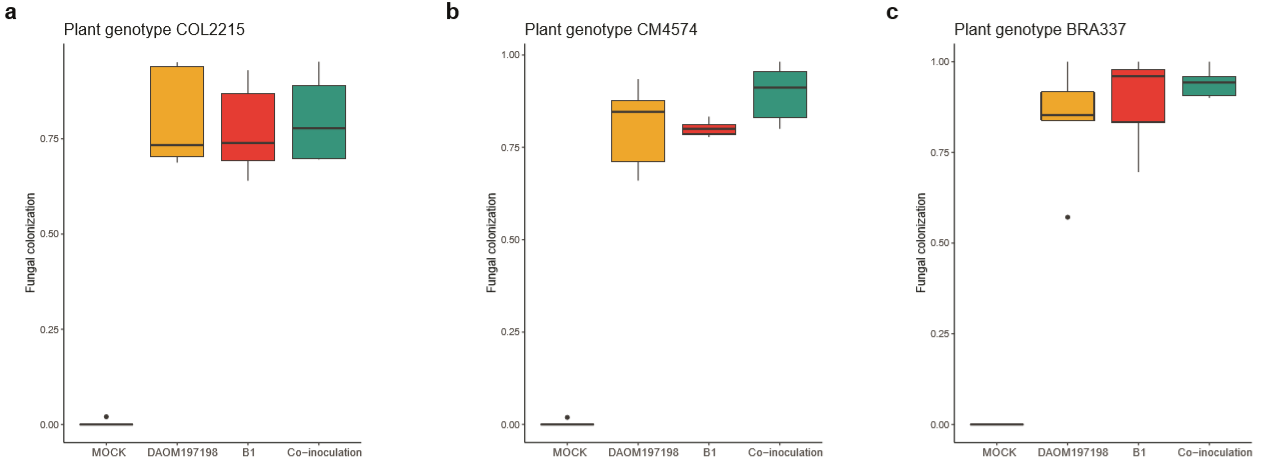
**

**Supplementary Fig. 5. Fungal colonization detected by the grid-intersect method in each treatment.** The Y axis describes the fungal colonization as a percentage of intersects with fungal structures detected / total number of intersects. Data is shown independently for **a** host plant genotype COL2215, **b** host plant genotype CM4574 and **c** host plant genotype BRA337. * denotes a statistically significant difference between a given pair of treatments.

**
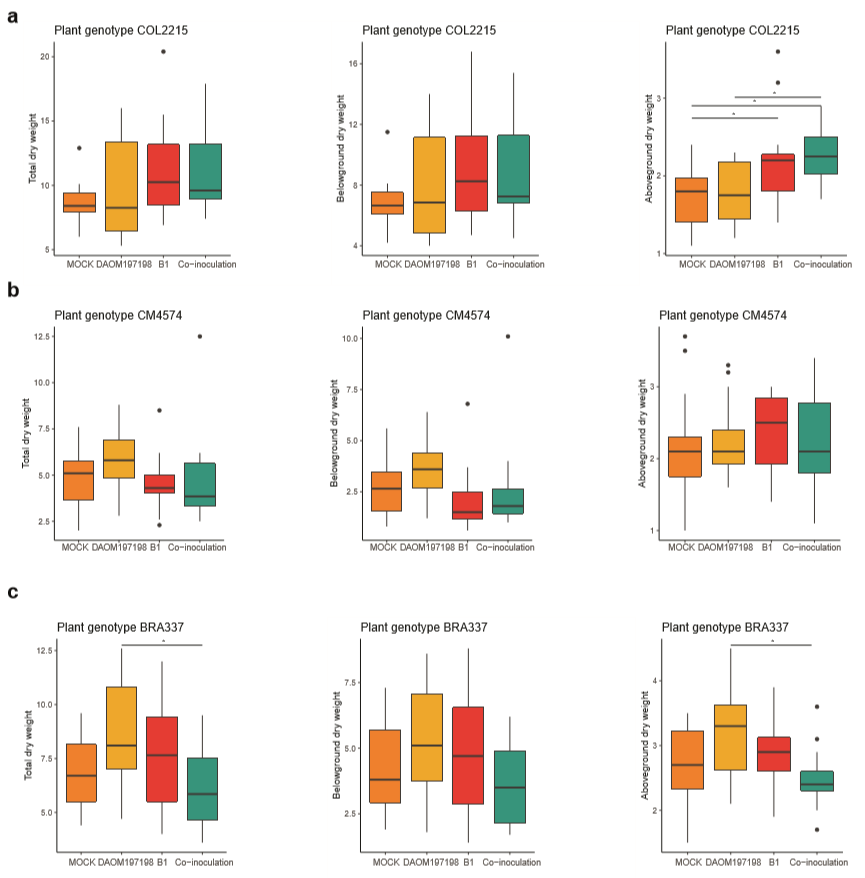
**

**Supplementary Fig. 6. Plant growth responses.** We show the total dry weight, belowground dry weight and aboveground dry weight of the different treatments of **a** host plant genotype COL2215, **b** host plant genotype CM4574 and **c** host plant genotype BRA337. * denotes a statistically significant difference between a given pair of treatments. The Y axis represent the weight in grams.

**
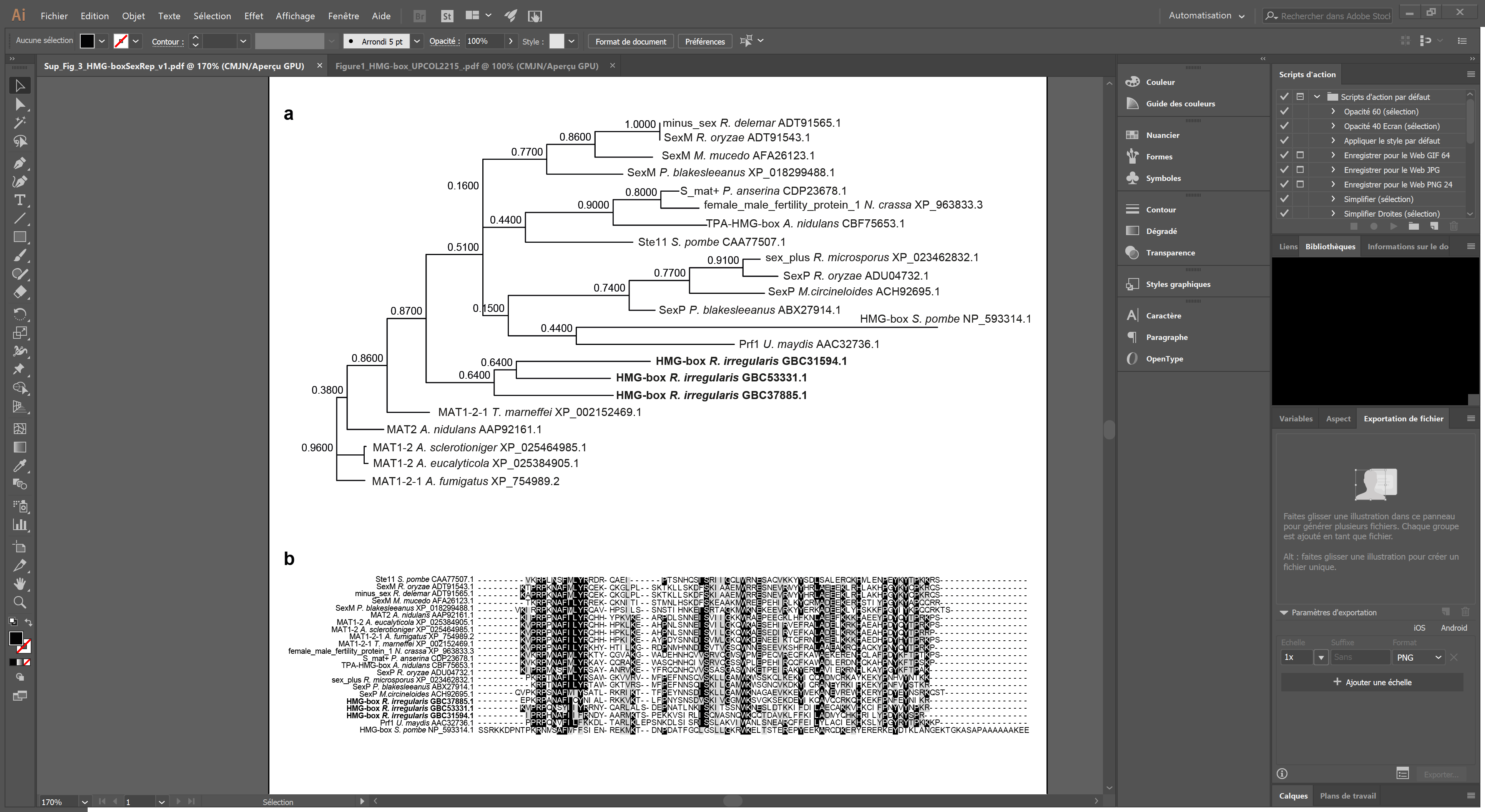
**

**Supplementary Fig. 7. Phylogenetic reconstruction of the HMG-boy domain and alignment of HMG-box genes in fungi. a** Maximum likelihood phylogenetic reconstruction of the HMG-box domain of HMG-box genes involved in sexual reproduction in fungi, including the three upregulated HMG-box genes observed in this study (shown in bold type). LG + G was selected as the substitution model and we performed 100 bootstraps. We show the accession numbers of the different HMG-box genes. **b** Alignment of the conserved domain of HMG-box genes involved in sexual reproduction in fungi including the three upregulated HMG-box genes observed in this study (shown in bold type).

**
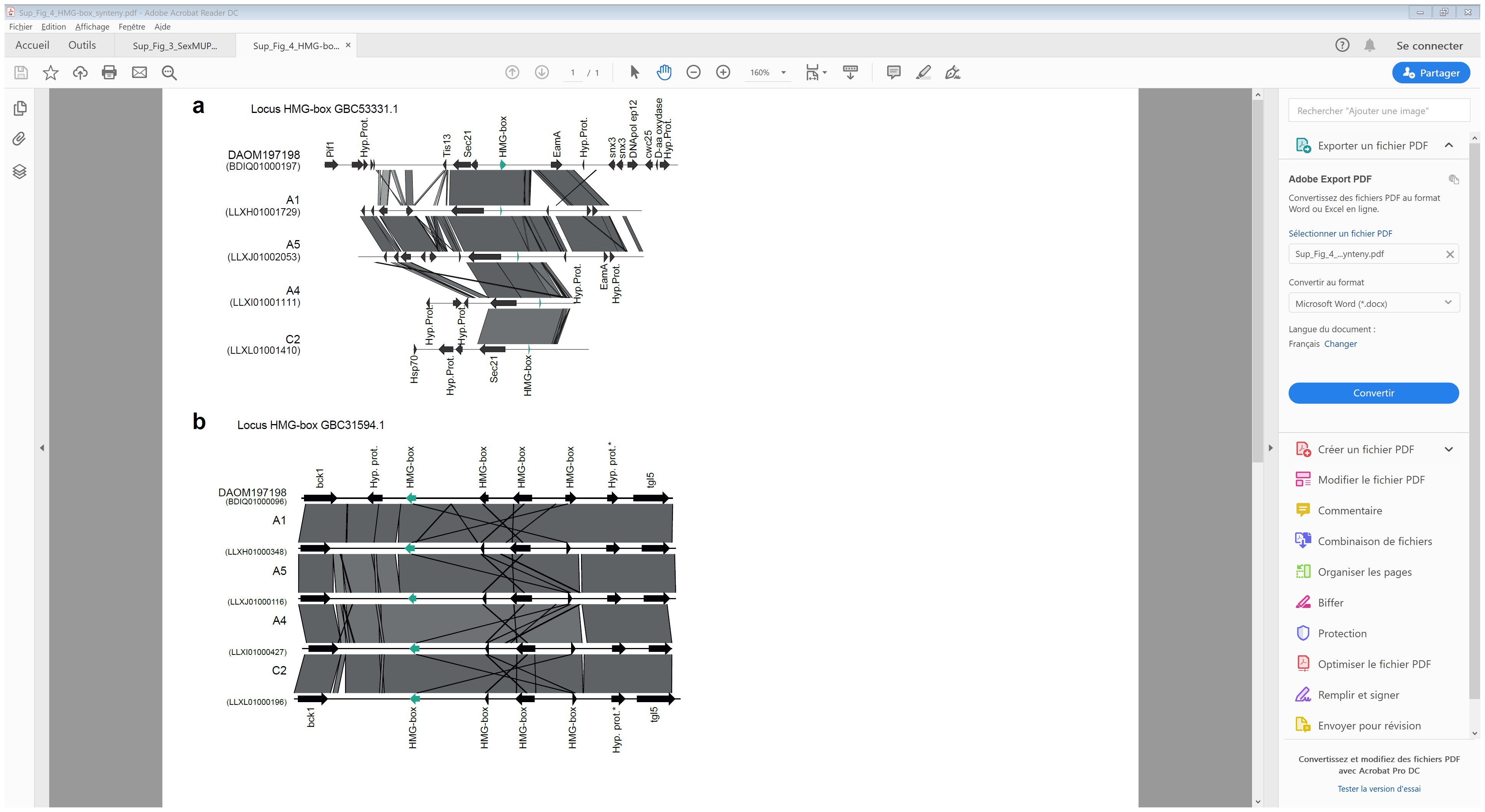
**

**Supplementary Fig. 8. Synteny plot of the contigs containing the induced HMG-box genes**. **a** GBC53331.1 and **b** GBC31594.1 in five genetically different *R. irregularis* isolates DAOM197198, A1, A5, A4 and C2. Grey lines linking the strains represent homologous regions among the isolates. The induced HMG-box gene is represented in green. *The fifth HMG-box gene in isolates DAOM197198 and C2 on the locus containing GBC31594.1 was not detected as an HMG-box domain because of an amino acid substitution in the N-terminal part of the sequence and is, therefore, labelled “hypothetical protein”.


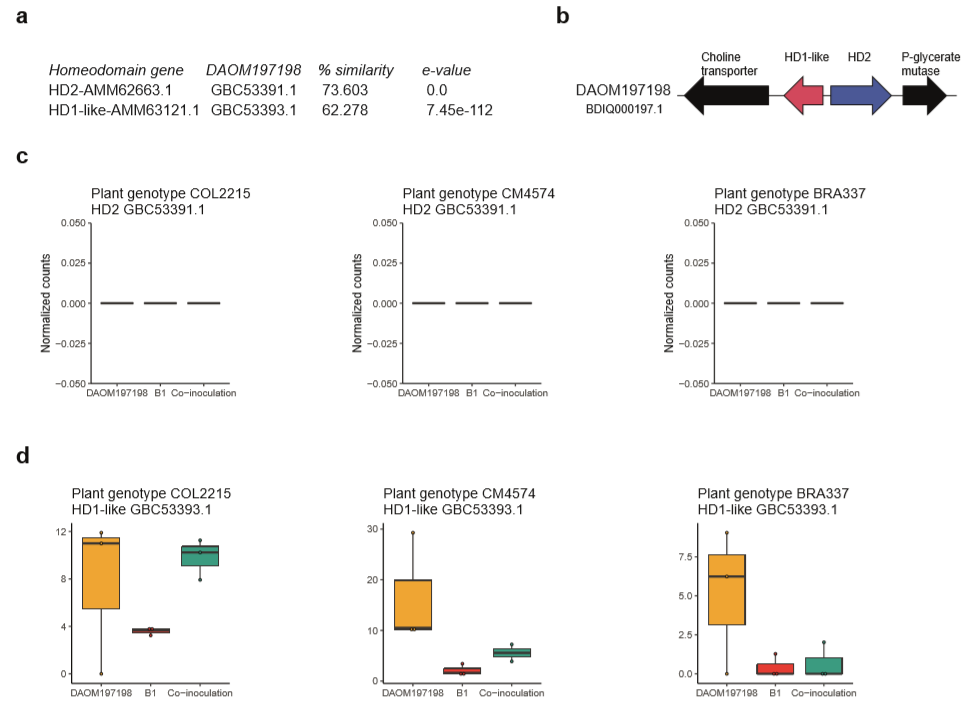


**Supplementary Fig. 9. Analysis of transcription of homeodomain HD2 and HD1-like genes encoded in the putative MAT-locus proposed by Ropars *et al.,* 2016 in three genetically different cassava cultivars (COL2215, CM4574 and BRA337).** **a** Blast homology of HD2 and HD1-like genes to the gene-prediction used in this study. **b** Gene organization of the putative MAT-locus in *R. irregularis* DAOM197198. **c** Gene transcription of HD2 and **d** HD1-like genes in single inoculations and the co-inoculation treatments of this study. We show the normalized counts per treatment.

**
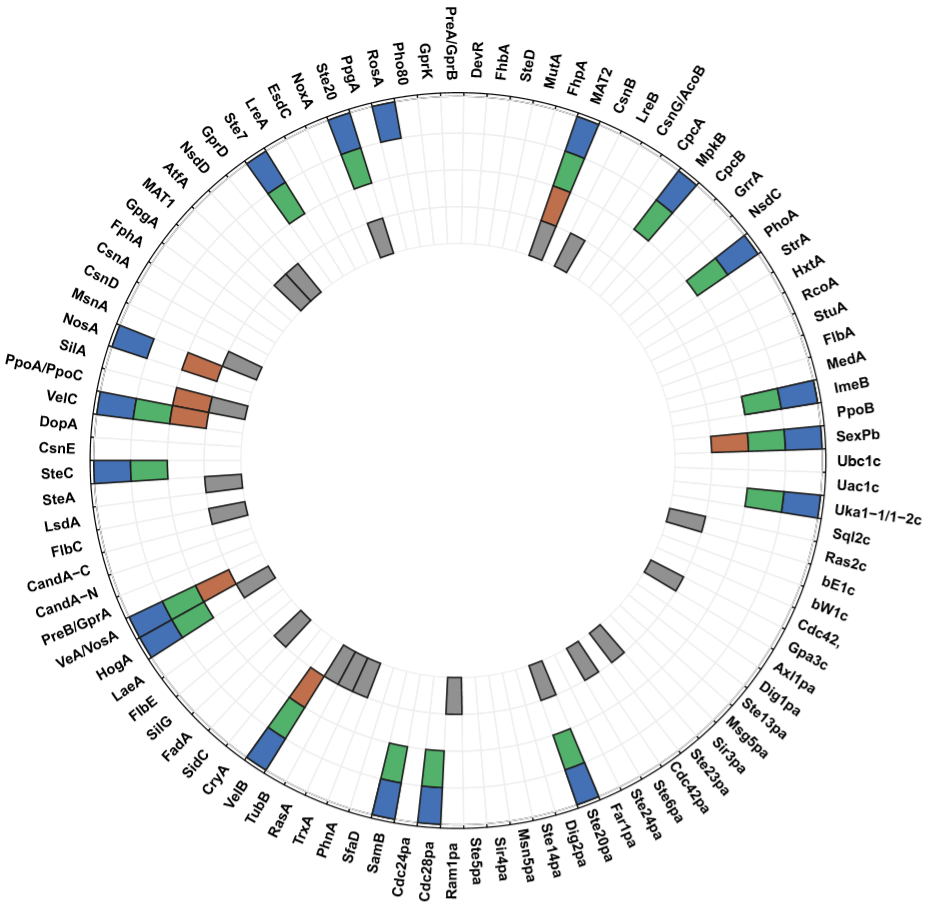
**

**Supplementary Fig. 10. Genes potentially involved in sexual reproduction in *R. irregularis*.** Homologs to reproductive proteins across a fungal gene set defined by Mondo *et al.,* 2018. We highlighted all blast hits to the reproductive proteins in this fungal gene list of *R. irregularis* genes that were significantly differentially transcribed in the co-inoculation treatment compared to the single-inoculations in host genotypes COL2215 (blue), CM4574 (green) and BRA337 (brown). As a comparison we show the genes that were differentially transcribed during confirmed sexual reproduction in the Mucoromycotina species *Rhizopus microsporus* [1] (grey). For comparative purposes, genes names were obtained and shown as in Mondo *et al.,* 2017. Consequently, gene names differ from the gene names described in this study.

**
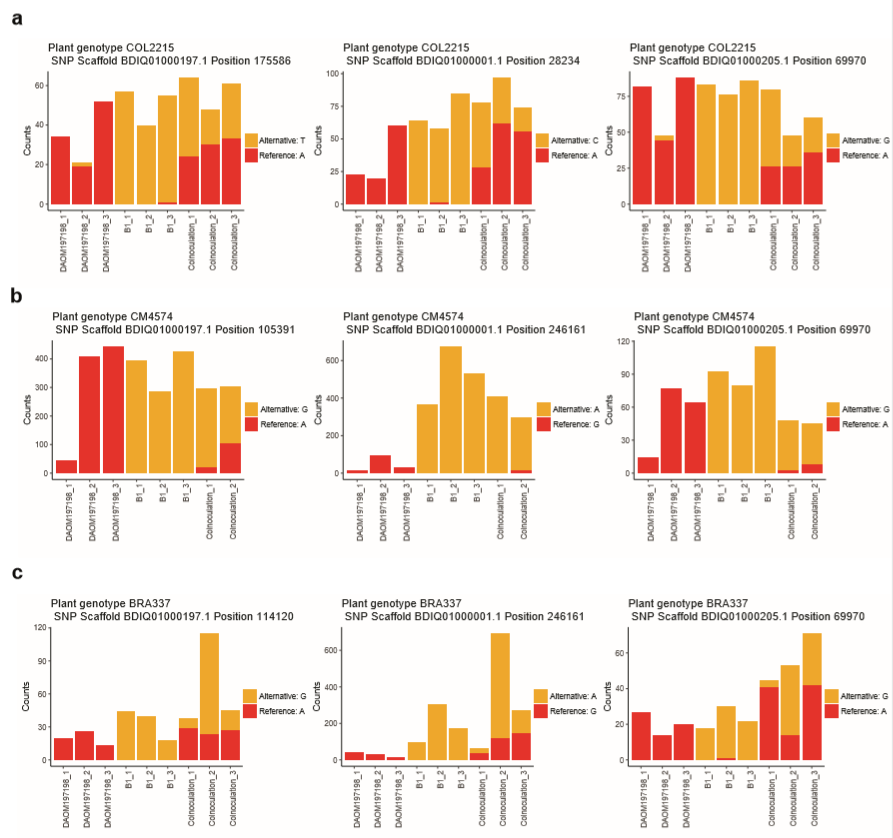
**

**Supplementary Fig. 11. Analysis of SNPs in RNA-seq data of three random individual positions.** Allele counts of the reference or alternative allele in each sample. **a** data from host plant genotype COL2215, **b** host plant genotype CM4574 and **c** host plant genotype BRA337.

Supplementary Notes

**Co-existence of genetically different *Rhizophagus irregularis* isolates induces genes involved in a putative fungal mating response**

Ivan D. Mateus*, Edward C. Rojas, Romain Savary, Cindy Dupuis, Frédéric G. Masclaux, Consolée Aletti & Ian R. Sanders*

**Supplementary Note 1.** The purpose of this study

The purpose of this study was to find out whether some genes were specifically expressed by the two genetically different *R. irregularis* isolates when they co-existed in the plant, and if so, which genes were expressed and did they point to any particular biological processes that could indicate an interaction between the two fungi. The experiment was performed in parallel with three genetically different host plant genotypes to evaluate whether or not the host plant genotype played a role in mediating the molecular interactions between two different AMF genotypes. To do this, the transcript sequences were first aligned to the host plant genome. Then the remaining unmapped reads were aligned to the *R. irregularis* genome, yielding a data set of *R. irregularis* specific transcripts (Supplementary file 1, Supplementary Figure 1b). We then compared fungal gene transcription profiles between the single inoculation and the co-inoculation treatments to detect genes specifically up- or down-regulated in co-inoculation treatments (Supplementary Figure 1c).

**Supplementary Note 2.** Preparation of greenhouse experiments with *in-vitro* plant and fungal material.

We micro-propagated cassava *in-vitro* using cuttings from lateral and apical meristems, approximately 1 cm long. Explants were grown on MS medium with 14 h daylight at 25°c in a plant growth chamber (Sanyo MLR-351 H). After 8 weeks of growth, plants were hardened-off in greenhouse conditions (28°C, 16 h daylight and 70% RH) for four weeks. The hardening substrate was an autoclaved mixture of perlite and peat moss (1:1). Hardened plants were transplanted to final steam sterilized (100°C, 25 min) substrate; comprising perlite, moss peat, inert clay and sand (1:1:1:1) (v/v). After inoculation, plants were grown in greenhouse conditions and watered regularly. We positioned the plants in a randomised block design in the greenhouse, where each block contained one replicate of each of the treatments. Additionally, we randomised the position of the blocks every 4 weeks in order to avoid block effects. We used *R. irregularis* isolates B1 and DAOM197198 to inoculate the plants. Spores from *in-vitro* culture split-plates [2] were extracted by dissolving the medium in a solution of citric acid (6%), and passed through a sieve (30 µm). We inoculated the plants with 300 spores of isolate B1 or with 300 spores of isolate DAOM197198 or with 150 spores of each isolate as a co-inoculation treatment. We performed the inoculation by suspending the spores in 10 ml of distilled water and applying it directly to the roots. The mock-inoculated control contained only 10 ml of distilled water.

**Supplementary Note 3.** Fungal colonization and plant growth responses

As described in Mateus *et al.,*[3] we measured all traits 18 weeks after inoculation. We randomly selected fine roots to measure overall fungal colonization. We cleared them with 10% KOH for 4 hrs, acidified them with HCl (1%) during 5 minutes and stained them with trypan blue (0.10% in a lactic acid-glycerol solution) overnight. Root colonization was determined using 5 replicates of each treatment by the grid line intersect method [4]. We separated the aboveground and belowground plant material and dried it for eight days at 72°C. We measured aboveground and belowground dry weight independently. We performed an ANOVA followed by a Tukey post-hoc test to test for significant statistical differences between the treatments. Each cultivar was analysed independently.

**Supplementary Note 4.** RNA extraction, library preparation, sequencing and bioinformatic analysis

After 18 weeks of growth in the greenhouse, we harvested the plants. We washed the roots and approximately 150 mg of fine roots were then randomly chosen from each plant for RNA extraction. We extracted RNA, made libraries and sequenced 3 replicates of each treatment of each cultivar separately. The RNA was extracted from the root tissue using the Maxwell plant RNA kit (Promega). We used the *TruSeq Stranded mRNA* Library Prep Kit, set B to make the library preparation. The libraries were sequenced using Illumina® Hi-seq 2500 100bp paired-end technology. Because of low library quality or contaminations, we excluded 1 mock-inoculated sample from cultivar COL2215 and 1 co-inoculated sample of cultivar CM4574.

After sequencing, we processed the raw sequences with Trim Galore v.0.6.0 with its default parameters to trim Illumina adapters [5] and to quality-filter the reads (https://github.com/FelixKrueger/TrimGalore). To differentiate between the cassava and AM fungal RNA-seq data sets, we aligned the total reads to the *M. esculenta* reference genome (*M. esculenta* V6.1,Phytozome V11) [6] with a two pass method with the STARstatic 2.4.0 alligner [7]. We discarded the reads mapping to the cassava genome and proceeded to map the unaligned reads, to the *R. irregularis* genome [8]. We only used the exclusive *R. irregularis*-mapping reads for further analyses. We then used featureCounts, from the subread package v1.6.0 [9], in order to produce tables of the number of counts per transcript per sample. Our analysis only considered reads uniquely assigned to each genetic feature and multiple assigned reads were discarded. We used the long-read genome assembly and annotation of isolate DAOM197198 (GCA_002897155.1) [8] to perform the analysis. We analysed 3 replicates for each treatment (DAOM197198: n=3, B1: n=3, co-inoculation: n=3) on plant genotype COL2215 and BRA337. We produced 3 replicates for all the treatments on cultivar CM4574, in exception of the co-inoculation treatment (n=2).

**Supplementary Note 5.** Similarity between the Mucormycotina MAT-locus and *R. irregularis* locus of GBC53331.1.

The *R. irregularis* locus and the MAT-locus of the other Mucoromycotina species shared the presence of the HMG-box and a membrane transporter (EamA in *R. irregularis* and TPT in Mucoromycotina). EamA and TPT domains are closely related as, EamA is proposed to be the ancestor domain of the TPT family [10] and be part of the same family domain: DMT (CL0184). However, while the MAT-locus in the Mucoromycotina species contains an RNA helicase, in the *R. irregularis* locus, this is replaced by a coatomer gamma subunit containing the N-terminal of an adaptin-conserved domain (pf01602).

**Supplementary Note 6.** Upregulated genes in host genotype COL2215 involved in different stages of the putative fungal mating response.

We identified a homolog of the *U. maydis* Aga1 gene (GBC40214.1), which is a cAMP-dependent protein-kinase involved in pheromone perception [11]. We also observed the specific upregulation of genes that are present in the MAT-locus of closely related Mucoromycotina species. In addition to the HMG-box genes, described above, we found two homologs of an RNA helicase (ecm32: GBC31744.1 and GBC46658.1) [12]. Two genes involved in mitogen-activated protein-kinase (MAPK) pathways were upregulated and which are relevant to four MAPK signalling cascades induced by the pheromone response. The gene STE20 (GBC47251.1) is activated directly in the pheromone pathway, the high osmolarity (HOG) and invasive filamentous growth pathways [13]. The gene Mkk2 (GBC27247.1) is involved in the protein-kinase C (PKC) cascade which plays a role in the cell wall integrity during the pheromone response [13]. In addition, to cell differentiation, exposure to pheromones can induce cell death [14]. We identified a Ca2+/calmodulin-dependent protein kinase (cmk1; GBC21696.1) which is involved in the cell survival to pheromones [15].

We also found two genes that are involved in the formation of mating tubes in other fungal species. We found a homolog (GBC27006.1) of the fuz1 gene in *U. maydis*, that is necessary for the formation of the mating tube [16]. We also detected the methionine transporter mup1 (GBC28192.1). Methionine transporters could have a role in mating as methionine is found to be relevant for the formation of mating tubes [17]. Furthermore, we found two genes involved in meiosis. We detected Rad53 (GBC47027.1), which has a role in signalling of unrepaired double-strand breaks during meiosis [18]. We also identified a homolog of IpI1 (GBC38036.1) which is involved in sister chromatid cohesion and spindle-pole body cohesion during meiosis [19]. In sexual sporulation, after completion of the meiosis cycle, the gene sps1 plays a role in the encapsulation of haploid nuclei within the spore wall [20]. In addition, the gene dit2 is involved in the formation of the outermost layer of spores [21]. We found homologs of both genes (sps1: GBC21972.1, dit2: GBC28793.1) in the set of genes that were differentially transcribed in the co-inoculation treatment compared to the single inoculations. We also detected homologs of genes veA (GBC19598.1) and skt5p (GBC21938.1) which are regulators during mating [22, 23]. Finally, we identified Aig1 (GBC10892.1), a guanine nucleotide-binding G-domain protein (G-protein). G-proteins are known pheromone receptors of fungal species [24]. Although we did not find a significant blast hit to the yeast GPA1 G-protein, a pheromone receptor [25], aig1 and gpa1 shared the P-loop_NTPase super family containing the guanine nucleotide-binding region. This suggests that gpa1 is a G-protein that could have a convergent role in pheromone perception.

**References**

1. Mondo SJ, Lastovetsky OA, Gaspar ML, Schwardt NH, Barber CC, Riley R, *et al.* Bacterial endosymbionts influence host sexuality and reveal reproductive genes of early divergent fungi. *Nat Commun* 2017; **8**: 1843.

2. Rosikiewicz P, Bonvin J, Sanders IR. Cost-efficient production of in vitro *Rhizophagus irregularis*. *Mycorrhiza* 2017; **27**: 477–486.

3. Mateus ID, Masclaux FG, Aletti C, Rojas EC, Savary R, Dupuis C, *et al.* Dual RNA-seq reveals large-scale non-conserved genotype × genotype-specific genetic reprograming and molecular crosstalk in the mycorrhizal symbiosis. *ISME J* 2019; **13**: 1226–1238.

4. Giovannetti M, Mosse B. An evaluation of techniques for measuring vesicular arbuscular mycorrhizal infection in roots. *New Phytol* 1980; **84**: 489–500.

5. Schmieder R, Lim YW, Rohwer F, Edwards R. TagCleaner: Identification and removal of tag sequences from genomic and metagenomic datasets. *BMC Bioinformatics* 2010; **11**: 341.

6. Bredeson J V, Lyons JB, Prochnik SE, Wu GA, Ha CM, Edsinger-Gonzales E, *et al.* Sequencing wild and cultivated cassava and related species reveals extensive interspecific hybridization and genetic diversity. *Nat Biotechnol* 2016; **34**: 562–570.

7. Dobin A, Davis CA, Schlesinger F, Drenkow J, Zaleski C, Jha S, *et al.* STAR: Ultrafast universal RNA-seq aligner. *Bioinformatics* 2013; **29**: 15–21.

8. Maeda T, Kobayashi Y, Kameoka H, Okuma N, Takeda N, Yamaguchi K, *et al.* Evidence of non-tandemly repeated rDNAs and their intragenomic heterogeneity in *Rhizophagus irregularis*. *Commun Biol* 2018; **1**.

9. Liao Y, Smyth GK, Shi W. FeatureCounts: An efficient general purpose program for assigning sequence reads to genomic features. *Bioinformatics* 2014; **30**: 923–930.

10. Västermark Å, Almén MS, Simmen MW, Fredriksson R, Schiöth HB. Functional specialization in nucleotide sugar transporters occurred through differentiation of the gene cluster EamA (DUF6) before the radiation of Viridiplantae. *BMC Evol Biol* 2011; **11**: 123.

11. Berndt P, Lanver D, Kahmann R. The AGC Ser/Thr kinase Aga1 is essential for appressorium formation and maintenance of the actin cytoskeleton in the smut fungus *Ustilago maydis*. *Mol Microbiol* 2010; **78**: 1484–1499.

12. Gryganskyi AP, Lee SC, Litvintseva AP, Smith ME, Bonito G, Porter TM, *et al.* Structure, function, and phylogeny of the mating locus in the *Rhizopus oryzae* complex. *PLoS One* 2010; **5**: 1–12.

13. Roberts CJ, Nelson B, Marton MJ, Stoughton R, Meyer MR, Bennett HA, *et al.* Signaling and circuitry of multiple MAPK pathways revealed by a matrix of global gene expression profiles. *Science* 2000; **287**: 873–880.

14. Severin FF, Hyman AA. Pheromone induces programmed cell death in *S. cerevisiae*. *Curr Biol* 2002; **12**: 233–235.

15. Moser MJ, Geiser JR, Davis TN. Ca2+-calmodulin promotes survival of pheromone-induced growth arrest by activation of calcineurin and Ca2+-calmodulin-dependent protein kinase. *Mol Cell Biol* 1996; **16**: 4824–4831.

16. Chew E, Aweiss Y, Lu C, Banuett F. Fuz1, a MYND domain protein, is required for cell morphogenesis in *Ustilago maydis*. *Mycologia* 2008; **100**: 31–46.

17. Fischer JA, McCann MP, Snetselaar KM. Methylation is involved in the *Ustilago maydis* mating response. *Fungal Genet Biol* 2001; **34**: 21–35.

18. Cartagena-Lirola H, Guerini I, Manfrini N, Lucchini G, Longhese MP. Role of the *Saccharomyces cerevisiae* Rad53 Checkpoint Kinase in Signaling Double-Strand Breaks during the Meiotic Cell Cycle. *Mol Cell Biol* 2008; **28**: 4480–4493.

19. Shirk K, Jin H, Giddings TH, Winey M, Yu HG. The Aurora kinase Ipl1 is necessary for spindle pole body cohesion during budding yeast meiosis. *J Cell Sci* 2011; **124**: 2891–2896.

20. Friesen H, Lunz R, Doyle S, Segall J. Mutation of the SPS1-encoded protein kinase of *Saccharomyces cerevisiae* leads to defects in transcription and morphology during spore formation. *Genes Dev* 1994; **8**: 2162–2175.

21. Briza P, Breitenbach M, Ellinger A, Segall J. Isolation of two developmentally regulated genes involved in spore wall maturation in *Saccharomyces cerevisiae*. *Genes Dev* 1990; **4**: 1775–1789.

22. Kim HS, Han KY, Kim KJ, Han DM, Jahng KY, Chae KS. The veA gene activates sexual development in *Aspergillus nidulans*. *Fungal Genet Biol* 2002; **37**: 72–80.

23. Trilla JA, Cos T, Duran A, Roncero C. Characterization of CHS4 (CAL2), a gene of *Saccharomyces cerevisiae* involved in chitin biosynthesis and allelic to SKT5 and CSD4. *Yeast* 1997; **13**: 795–807.

24. Kurjan J, Hirsch JP, Dietzel C. Mutations in the guanine nucleotide-binding domains of a yeast G-protein confer a constitutive or uninducible state to the pheromone response pathway. *Genes Dev* 1991; 475–483.

25. Zhou J, Arora M, Stone DE. The yeast pheromone-responsive Gα protein stimulates recovery from chronic pheromone treatment by two mechanisms that are activated at sistinct levels of stimulus. *Cell Biochem Biophys* 1999; **30**: 193–212.
